# MAP4K2 suppresses antitumor immunity in a pancreatic cancer model by promoting Treg differentiation

**DOI:** 10.1172/JCI196379

**Published:** 2026-01-29

**Authors:** Huai-Chia Chuang, Chia-Wen Wang, Chia-Hsin Hsueh, Yu-Zhi Xiao, Ching-Yi Tsai, Pu-Ming Hsu, Evelyn L. Tan, Hsien-Yi Chiu, Tse-Hua Tan

**Affiliations:** 1 Immunology Research Center, National Health Research Institutes, Zhunan, Taiwan.; 2 Department of Dermatology, National Taiwan University Hospital Hsin-Chu Branch, Hsinchu, Taiwan.

**Keywords:** Cell biology, Immunology, Oncology, Protein kinases, Signal transduction, Tregs

## Abstract

MAP kinase kinase kinase kinase (MAP4K) family kinases are key kinases for T cell–mediated immune responses; however, in vivo roles of MAP4K2 in immune regulation remain unclear. Using T cell–specific *Map4k2* conditional knockout (T-*Map4k2* cKO) mice, scRNA-seq, and mass spectrometry analysis, we found that MAP4K2 interacted with DDX39B, induced forkhead box protein P3 *(FOXP3)* gene expression, and promoted Treg differentiation. Mechanistically, MAP4K2 directly phosphorylated the DEAD box protein DDX39B, leading to DDX39B nuclear translocation and subsequent *Foxp3* RNA splicing. MAP4K2-induced *FOXP3* mRNA levels were abolished in *DDX39B* knockout T cells. Furthermore, T-*Map4k2* cKO mice displayed the reduction of Treg population and the sustained inflammation during remission phase of EAE autoimmune disease model. Remarkably, the anti–PD-1 immunotherapeutic effect on pancreatic cancer was significantly improved in T-*Map4k2* cKO mice, Treg-specific *Map4k2*-deficient mice, adoptively transferred chimeric mice, or MAP4K2-inhibitor–treated mice. Consistently, scRNA-seq analysis of patients with pancreatic cancer showed increased *MAP4K2* levels in infiltrating Treg cells. Collectively, MAP4K2 promotes Treg differentiation by inducing DDX39B nuclear translocation, leading to the attenuation of antitumor immunity.

## Abstract 2

MAP4K2 induces Treg differentiation via DDX39B. MAP4K2 suppresses antitumor immunity in pancreatic cancer by promoting Treg differentiation.

## Graphical abstract

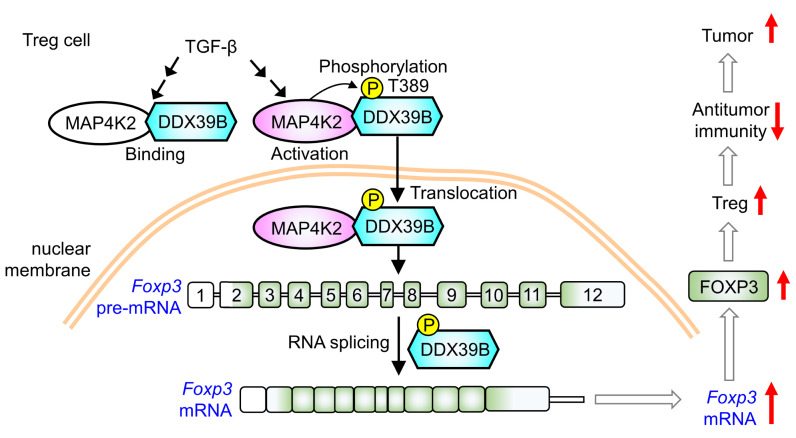

## Introduction

Regulatory T (Treg) cells suppress excessive innate or adaptive immune responses by modulating the activity or proliferation of multiple immune cells, including CD4^+^ T helper cells, CD8^+^ cytotoxic T cells, B cells, and dendritic cells (1, 2). Forkhead box protein P3 (FOXP3) is the master transcription factor that controls the differentiation and function of Treg cells. Downregulation or mutation of *FOXP3* results in impaired Treg development and uncontrolled immune responses, leading to the induction of autoimmune diseases, including multiple sclerosis, systemic lupus erythematosus, rheumatoid arthritis, and lethal fulminant systemic autoinflammation (IPEX syndrome) (1–3). Besides downregulation of FOXP3 levels, increase of *FOXP3* alternative spliced isoforms (e.g., *FOXP3*-ΔExon 2 or *FOXP3*-ΔExon 7) occurs in patients of autoimmune diseases (4, 5). Proliferation and suppressive functions of murine Treg cells are affected by *Foxp3* truncated isoforms (6). Thus, the regulation of *Foxp3* RNA splicing plays a critical role in maintaining Treg development and function.

MAP kinase kinase kinase kinase (MAP4K) family kinases are key kinases for T cell–mediated immune responses (7–9). MAP4K2 (also named germinal center kinase or GCK) (10), a serine threonine kinase, belongs to the mammalian Ste20-like kinase. MAP4K2-mediated autophagy contributes to the development of head and neck cancer (11). MAP4K2 also induces cell growth and cell proliferation of multiple myeloma by inhibiting apoptosis (12). MAP4K2 shares 57.4% and 46.2% amino acid identity with MAP4K3 (GLK) and MAP4K1 (HPK1) (13–16), which positively and negatively regulate T cell–mediated immune responses, respectively (7, 8). It is likely that MAP4K2 also regulates T cell functions. The in vivo role of MAP4K2 has been characterized using whole-body *Map4k2* knockout (KO) mice; however, the original publication has been retracted (16, 17). To date, the in vivo role of MAP4K2 in immune responses remains unclear. Here, we reported that MAP4K2 induces *Foxp3* RNA splicing by phosphorylating DDX39B, contributing to the induction of mature *Foxp3* mRNA and subsequent Treg cell differentiation.

## Results

**Figure 1:**
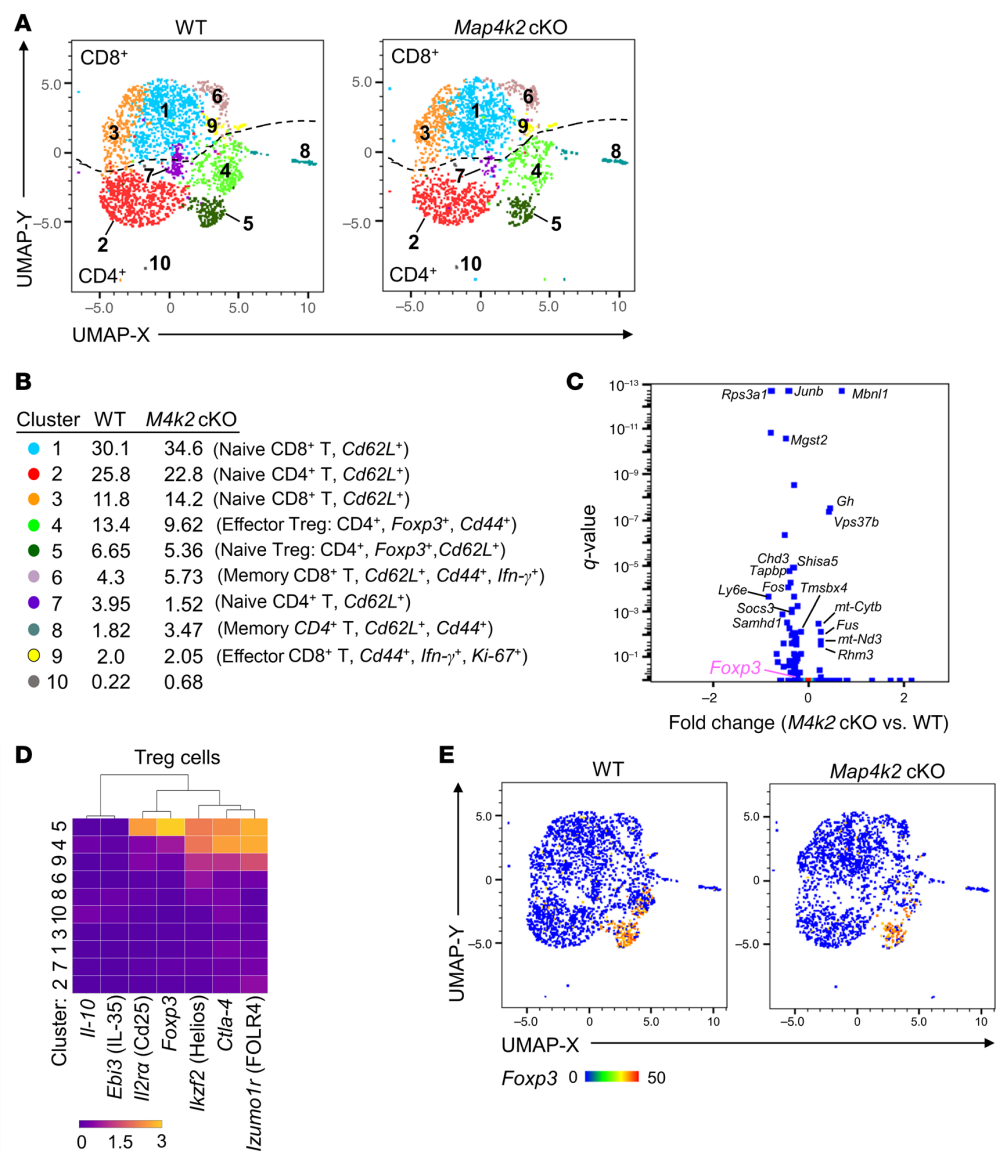
T*-Map4k2* cKO mice display a reduction of Treg population. (**A** and **B**) Single-cell RNA sequencing data showed the distribution (**A**) and classification (**B**) of pooled splenic and lymph node T cells from T*-Map4k2* *(M4k2*) cKO and WT mice. Data are presented in Uniform Manifold Approximation and Projection (UMAP). (**C**) The volcano plot of the selected differentially expressed genes in the T cells isolated from the spleen and lymph nodes of T*-Map4k2* cKO and WT mice. Q-value was determined using Fisher’s exact test. (**D**) Gene-expression heatmap of the Treg signature genes in each cluster compared with all other clusters. (**E**) Single-cell *Foxp*3 expression in T*-Map4k2* cKO and WT T cells. *M4k2* cKO, T cell–specific *Map4k2* conditional knockout (*Map4k2*^fl/fl^*;Cd4*-Cre); WT, WT *(Map4k2*^fl/fl^).

To study the in vivo role of MAP4K2 in T cell function, we generated T cell–specific *Map4k2* conditional knockout (T-*Map4k2* cKO, *Map4k2*^fl/fl^;*Cd4*-Cre) mice by crossing *Map4k2*-floxed mice to *Cd4*-Cre transgenic mice. The MAP4K2 levels were successfully abolished in T cells of T-*Map4k2* cKO mice (Supplemental Figure 1; supplemental material available online with this article; https://doi.org/10.1172/JCI196379DS1). The development of CD4^+^ and CD8^+^ T cell was normal in 4-week-old T-*Map4k2* cKO mice (Supplemental Figure 2, A–D). The thymic negative selection was also unaffected by *Map4k2* deficiency (Supplemental Figure 2B). Notably, the population of regulatory T cells (Treg, CD4^+^FOXP3^+^) was decreased in the spleen and lymph nodes, but not the thymus, of T-*Map4k2* cKO mice (Supplemental Figure 2, E–G). To study the T cell profiles and potential immune phenotypes of T-*Map4k2* cKO mice, T cells from the spleen and lymph nodes of T-*Map4k2* cKO mice were isolated and subjected to single-cell RNA-seq (scRNA-seq) analysis. Dimensionality reduction and clustering analyses showed 10 clusters of T cells from WT and T-*Map4k2* cKO mice (Figure 1, A and B, and Supplemental Figure 3A). Notably, expression levels of 34 genes were decreased, while 7 other genes were increased, in T cells of T-*Map4k2* cKO mice (Figure 1C). Kyoto Encyclopedia of Genes and Genomes (KEGG) pathway analyses revealed that these 34 downregulated genes in T-*Map4k2* cKO T cells belong to IFN-α/β signaling, IL-6 signaling, and regulation of antigen processing and presentation (Supplemental Figure 3B). Interestingly, among the differentially expressed genes in T-*Map4k2* cKO T cells, the Treg master transcription factor *Foxp3* was significantly decreased (Figure 1C, *P* = 0.000015). The *Foxp3*-positive T cells were mainly in the CD4^+^ Cluster no. 4 and Cluster no. 5, but neither in other CD4^+^ Clusters nor in CD8^+^ Clusters (Figure 1, D and E). The Cluster no. 4 and Cluster no. 5 were effector Treg and naive Treg cells, respectively (Figure 1B and Supplemental Figure 3A). The cell numbers of *Foxp3*-positive T cells were decreased in T-*Map4k2* cKO mice compared with those of WT mice (Figure 1E). Consistent with the notion that FOXP3 suppresses the *IFN-γ* transcription (18), *Map4k2*-deficient Treg cells displayed a modest increase of IFN-γ transcripts compared with WT Treg cells (fold change: 1.266). The mRNA levels of Treg signature genes, such as cytotoxic T-lymphocyte-associated protein 4 (*Ctla-4*), programmed death-1 (*Pd-1*), *Il-10*, and *Il2ra* (*Cd25*) were also significantly decreased in the T cells of T-*Map4k2* cKO mice (Supplemental Figure 3C). The decrease of Treg markers (CTLA-4, Helios, and CD25) and effector Treg markers (CD44 and CD62L) in the T cells of T-*Map4k2* cKO mice was confirmed by flow cytometry (Supplemental Figure 3, D and E). Notably, CD25 protein levels in the Treg (CD4^+^FOXP3^+^) cells of T-*Map4k2* cKO mice were comparable with those of WT mice (Supplemental Figure 3E). Interestingly, *Map4k2* mRNA levels in WT T cells were correlated with *Foxp3* levels (Supplemental Figure 4, A and B). Consistently, Human Protein Atlas (HPA) database showed that *MAP4K2* transcripts in Treg subset were more than those in naive CD4 T, naive CD8 T, memory CD4 T, memory CD8 T, and mucosal-associated invariant T cells (Supplemental Figure 4C). MAP4K2 protein levels were increased in Treg cells compared with other T cell subsets (Supplemental Figure 4D); the low MAP4K2 levels in Th1/Th2/Th17 cells also suggest that TCR stimulation would not induce MAP4K2 expression. Furthermore, overexpression of MAP4K2 induced FOXP3 protein levels in murine primary T cells and human peripheral blood T cells (Supplemental Figure 4, E and F). These results suggest that MAP4K2 positively regulates FOXP3 levels and Treg differentiation, especially in effector Treg cells.

**Figure 2:**
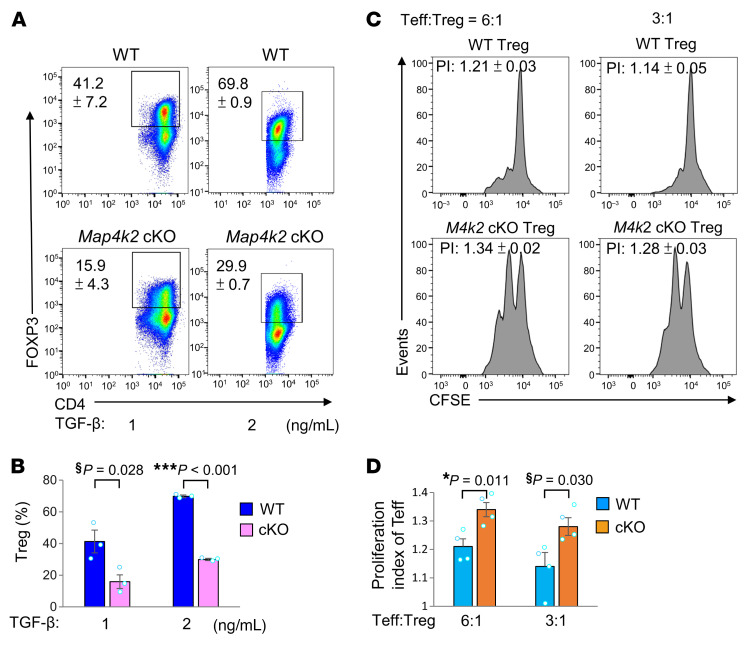
Treg differentiation and Treg suppressive function are attenuated by *Map4k2* knockout. (**A** and **B**) Flow cytometry analyses of in vitro differentiated Treg (CD4^+^FOXP3^+^) cells induced with 1 or 2 ng/mL TGF-β using splenic T cells isolated from 8-week-old mice (**A**). Mean ± SEM of 3 independent experiments are shown in the panel (**B**). (**C** and **D**) Suppression of CSFE-labeled effector T (T_eff_) cells by the Treg cells isolated from 8-week-old T*-Map4k2* cKO and WT mice (**C**). Mean ± SEM of 3 independent experiments are shown in the panel (**D**). *M4k2* cKO, T cell–specific *Map4k*2 conditional knockout *(Map4k2*^fl/fl^*;Cd4*-Cre); WT, WT *(Map4k2*^fl/fl^); PI, proliferation index. ^§^*P* value < 0.05; (1-tailed Student’s *t* test); *P < 0.05; ***P < 0.001 (2-tailed Student’s *t* test).

To study the effect of MAP4K2 on Treg differentiation, in vitro T cell differentiation was performed using splenic T cells of WT mice and T-*Map4k2* cKO mice. Interestingly, in vitro Treg differentiation of T-*Map4k2* cKO T cells was reduced compared with WT T cells upon TGF-β stimulation (Figure 2, A and B), while in vitro differentiation of Th1, Th2, and Th17 was unaffected by T-*Map4k2* cKO (Supplemental Figure 5, A–H). Next, Treg suppressive function was determined by coculturing of effector T (T_eff_) cells and Treg cells. The proliferation of CFSE-labeled WT T_eff_ cells was suppressed by WT Treg cells, whereas the proliferation of WT T_eff_ cells was not inhibited by T-*Map4k2* cKO Treg cells (Figure 2, C and D). In addition, the T cell proliferation of effector T cells from T-*Map4k2* cKO mice were comparable with that of WT mice (Supplemental Figure 5I). Collectively, the data suggest that MAP4K2 is required for Treg differentiation and suppressive function.

**Figure 3:**
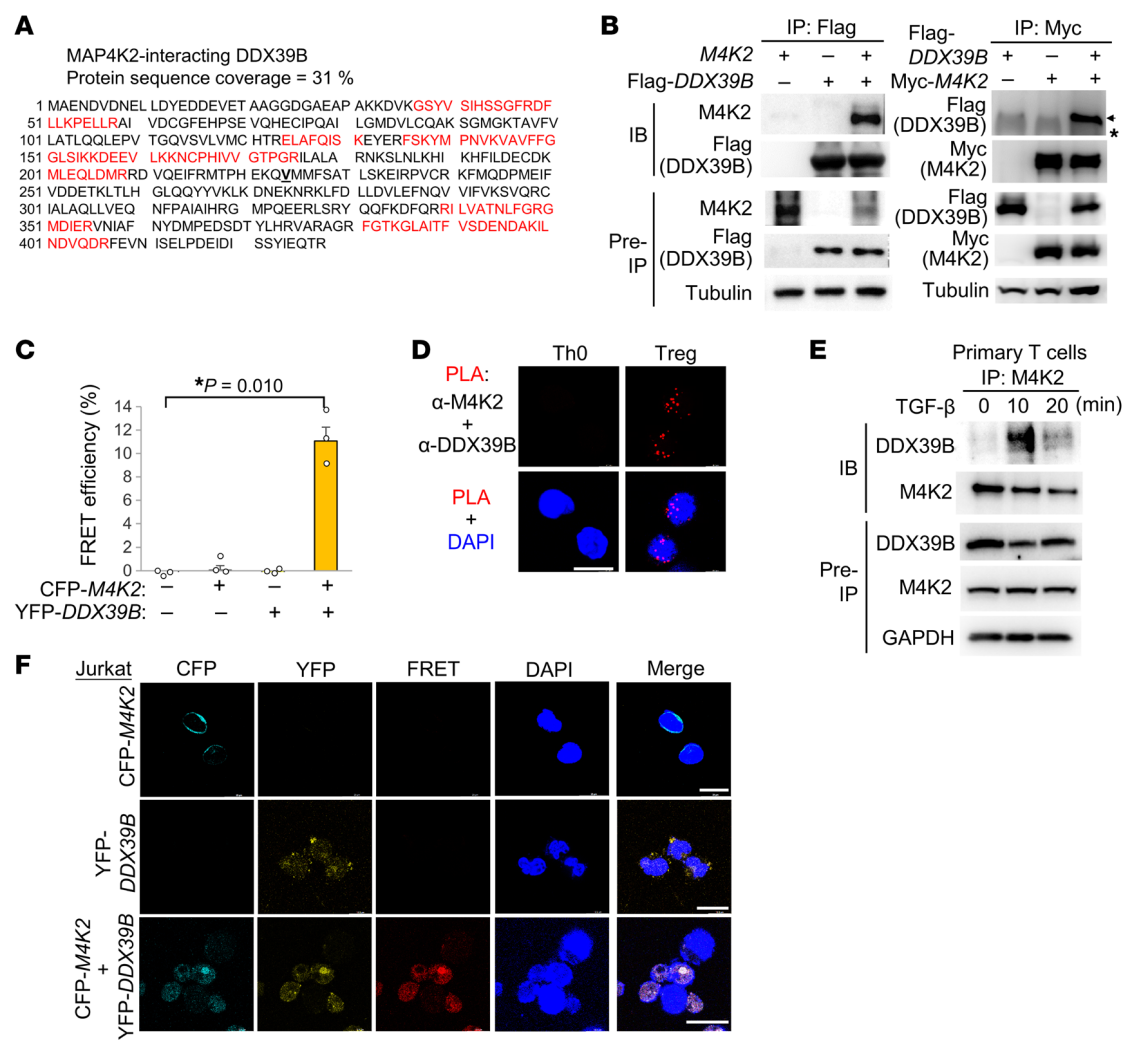
MAP4K2 directly interacts with DDX39B in T cells. (**A**) The detected peptide sequences (red color) of the endogenous DDX39B proteins by mass spectrometry–based proteomics analyses using the Myc-tagged MAP4K2 immunocomplexes isolated from Jurkat T cells stimulated with anti-CD3 antibody. (**B**) Reciprocal immunoprecipitation of Flag-tagged MAP4K2 (M4K2) with Myc-tagged DDX39B proteins from lysates of HEK293T cells cotransfected with Flag*-MAP4K2* and Myc*-DDX39B* plasmids. Arrowhead indicates the Flag-tagged DDX39B protein; asterisk indicates heavy-chain signals. (**C**) FRET analysis of HEK293T cells transfected with indicated plasmids encoding CFP-fused MAP4K2 or YFP-fused DDX39B proteins. Means ± SEM are shown. *n* = 3. (**D**) Confocal microscopy of PLA signals for the interaction between the endogenous MAP4K2 and DDX39B proteins in in vitro differentiated Treg cells. For PLA, red dots represent direct interaction signals. Scale bars: 10 μm. (**E**) Co-IP of the endogenous MAP4K2 and DDX39B proteins from lysates of murine primary T cells stimulated with TGF-β (125 ng/mL). (**F**) Confocal microscopy of CFP-fused MAP4K2 protein, YFP-fused DDX39B protein, FRET, and DAPI in transfected Jurkat T cells. Merge denotes the merge of all 4 color images. Scale bars: 25 μm. *P < 0.05 (2-tailed Student’s *t* test). Data shown (**B** and **D**–**F**) are representative of 3 independent experiments.

To investigate the mechanism of FOXP3 induction by MAP4K2, we studied whether the methylation of Treg-specific demethylated region (TSDR) is regulated by MAP4K2. The pyrosequencing data showed that TSDR methylation was unaffected in Treg (CD4^+^CD25^+^) cells of T-*Map4k2* cKO mice (Supplemental Figure 6). Next, to identify the MAP4K2-targeted molecules, MAP4K2-interacting proteins were searched by mass spectrometry analysis of anti-MAP4K2 immunocomplexes isolated from Jurkat T cells upon TCR stimulation. The DEAD box protein DDX39B, a known Treg regulator, was identified as a TCR-signaling–inducible MAP4K2-interacting protein (Figure 3A). DDX39B activates *Foxp3* RNA splicing and induces Treg population (19); therefore, we studied whether MAP4K2 induces FOXP3 levels through DDX39B. First, the physical interaction between MAP4K2 and DDX39B was confirmed by reciprocal coimmunoprecipitation (Figure 3B). To further demonstrate the direct interaction of MAP4K2 with DDX39B, cyan fluorescent protein–*MAP4K2* (CFP-*MAP4K2)* and yellow fluorescent protein–*DDX39B* (YFP-*DDX39B*) plasmids were transfected into HEK293T cells, followed by fluorescence resonance energy transfer (FRET) analysis. The FRET signals, denoting a protein-protein interaction (<10 nm), were detectable in CFP-*MAP4K2* and YFP-*DDX39B* plasmids-cotransfected HEK293T cells (Figure 3C), while CFP-*MAP4K2* or YFP-*DDX39B* alone did not show any FRET signals. Moreover, the interaction (<40 nm) between the endogenous MAP4K2 and DDX39B proteins in murine Treg cells, but not Th0 cells, was confirmed by in situ proximity ligation assay (PLA) (Figure 3D). Treg differentiation in vitro is induced by TGF-β and IL-2 (1). Consistently, we found that MAP4K2 kinase activity was induced by TGF-β treatment in a dose-dependent manner (Supplemental Figure 7A). Moreover, the interaction between the endogenous MAP4K2 and DDX39B proteins in murine primary T cell was induced upon TGF-β stimulation (Figure 3E). Notably, MAP4K2 protein levels in T cells were not regulated by TGF-β signaling (Figure 3E). In addition, the interaction between MAP4K2 and DDX39B was unaffected by the loss of MAP4K2 kinase activity (Supplemental Figure 7B). These results suggest that TGF-β signaling induces a direct interaction between MAP4K2 and DDX39B in Treg cells. Notably, the nuclear translocation of MAP4K2 and DDX39B proteins was induced in CFP-*MAP4K2* and YFP-*DDX39B* plasmids-cotransfected Jurkat T cells (Figure 3F), whereas MAP4K2 or DDX39B protein mainly localized to the cytoplasm of single plasmid–transfected Jurkat T cells (Figure 3F). It is likely that the interaction between MAP4K2 and DDX39B also facilitates their nuclear translocation.

**Figure 4:**
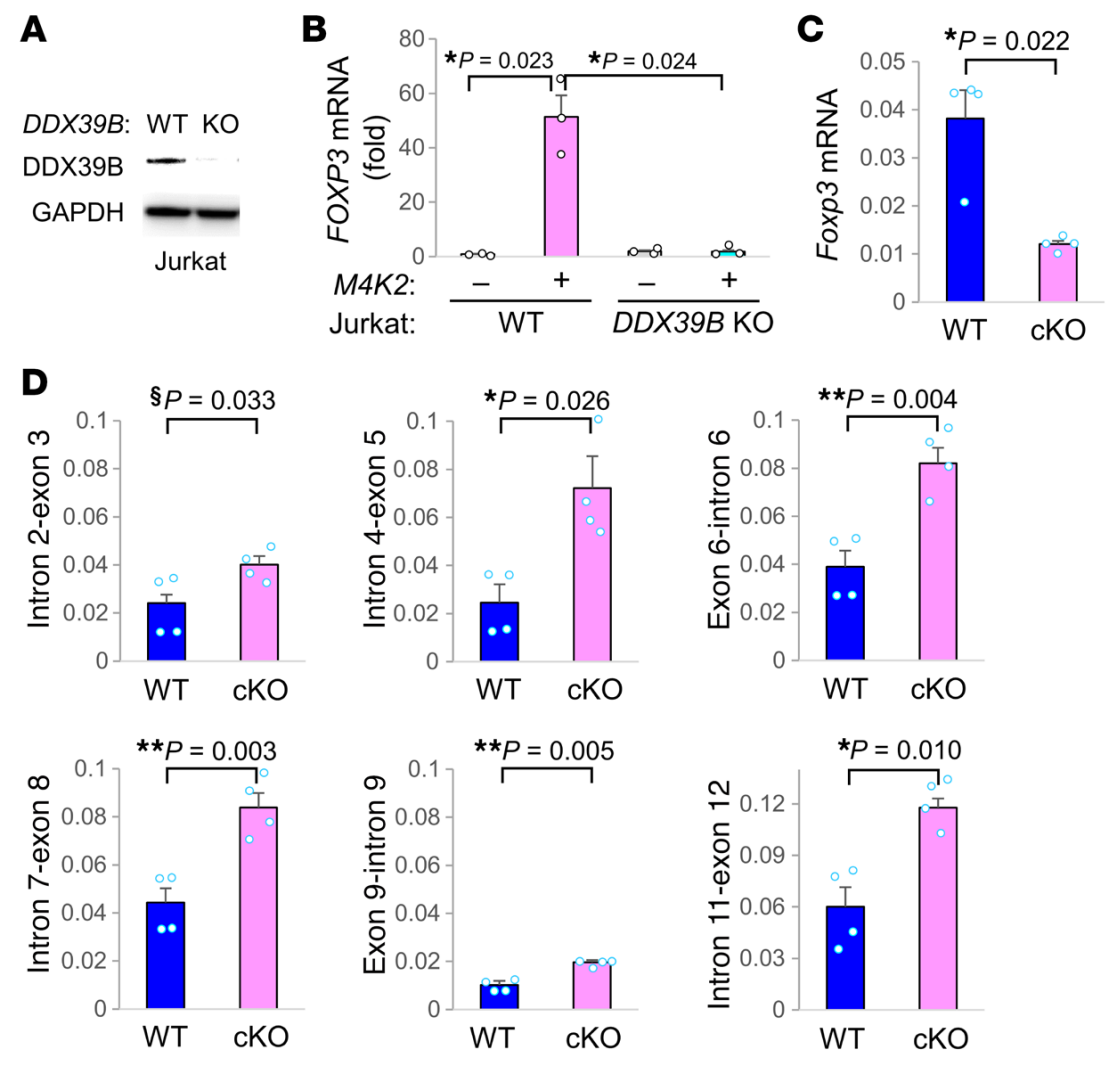
MAP4K2 promotes *Foxp3* RNA splicing through DDX39B. (**A**) Deficiency of DDX39B proteins in *DDX39B* knockout (KO) Jurkat T cells was confirmed by immunoblotting (upper panel). (**B**) Real-time PCR of *FOXP3* mRNA levels in WT or *DDX39B*-KO Jurkat T cells transfected with Myc*-MAP4K2* *(M4K2*) plasmid. The mRNA levels of *FOXP3* were normalized to *GAPDH* mRNA levels. Results (mean ± SEM, *n* = 3) are presented relative to those of vector controls. (**C** and **D**) Real-time PCR of *Foxp3* mRNA levels (**C**) and *Foxp3* pre-mRNA (individual junction regions across introns-exons) levels (**D**) in in vitro differentiated Treg cells of T*-Map4k2* cKO (cKO) and WT mice. The *Foxp3* mRNA levels were normalized to *Gapdh* mRNA levels (**C**); individual *Foxp3* pre-mRNA levels were normalized to total *Foxp3* mRNA levels (**D**). Means ± SEM are shown. *n* = 3. ^§^*P* < 0.05; (1-tailed Student’s *t* test); *P < 0.05; **P < 0.01 (2-tailed Student’s *t* test).

To validate the role of DDX39B in MAP4K2-mediated increase of *Foxp3* mRNA levels in T cells, *DDX39B* knockout (KO) Jurkat cells were generated by the CRISPR-Cas9 gene targeting approach (Figure 4A). *FOXP3* mRNA levels were significantly induced by MAP4K2 overexpression in Jurkat cells, while MAP4K2-induced *FOXP3* mRNA levels were abolished by *DDX39B* knockout (Figure 4B). *Foxp3* mRNA levels of peripheral Treg cells were significantly decreased in T-*Map4k2* cKO mice compared with those WT mice (Figure 4C). Next, the DDX39B-mediated *Foxp3* RNA splicing in peripheral Treg cells of T-*Map4k2* cKO mice was evaluated. The ratios of individual introns of *Foxp3* pre-mRNA to *Foxp3* mRNA levels were increased in T-*Map4k2* cKO Treg cells compared with WT Treg cells (Figure 4D). The data showed that *Foxp3* mRNA splicing in Treg cells was attenuated by *Map4k2* conditional knockout. The enhanced *Foxp3* pre-mRNA levels in T-*Map4k2* cKO Treg cells may be the reason why the fold change of *Foxp3* levels in scRNA-seq (Figure 1C) was underestimated, as scRNA-seq analysis would detect both mRNAs and pre-mRNAs. Collectively, these results suggest that MAP4K2 interacts with DDX39B and subsequently induces *Foxp3* mRNA splicing.

**Figure 5:**
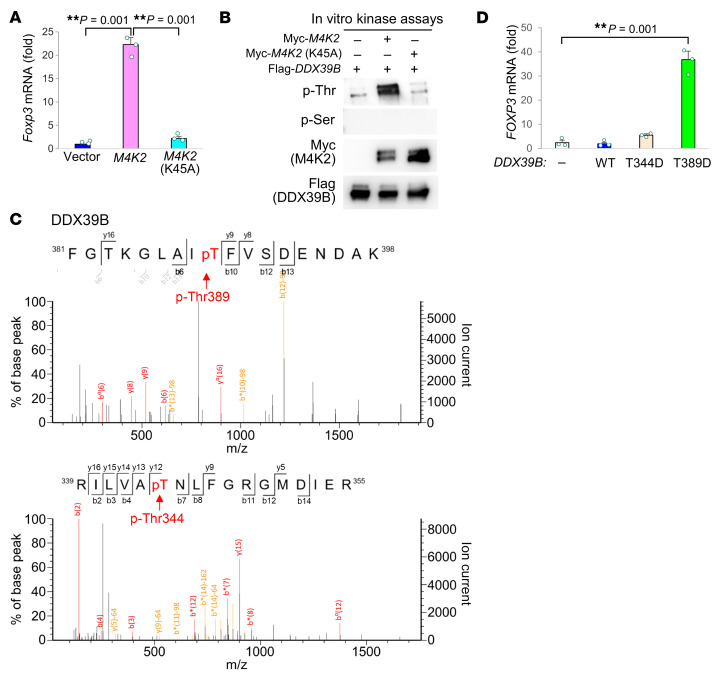
MAP4K2 directly phosphorylates DDX39B at Thr389 residue. (**A**) Real-time PCR of *FOXP3* mRNA levels in Jurkat T cells transfected with either Myc*-MAP4K2* (M4K2) WT or kinase-dead (K45A) plasmid. The mRNA levels of *FOXP3* were normalized to *GAPDH* mRNA levels. Results (mean ± SEM, *n* = 3) are presented relative to those of vector controls. (**B**) In vitro kinase assays of purified Myc-tagged MAP4K2 WT or kinase-dead (K45A) mutant proteins, using purified Flag-tagged DDX39B proteins as substrates. Phosphorylation of DDX39B was determined by immunoblotting analyses using anti-pan-phospho-serine (p-Ser) and anti-pan-phospho-threonine (p-Thr) antibodies. (**C**) Mass spectrometry analysis of the tryptic peptides from the DDX39B proteins phosphorylated by MAP4K2 to identify the peptide containing phosphorylated Thr389 or Thr344 residue on DDX39B. (**D**) Real-time PCR of *FOXP3* mRNA levels in Jurkat T cells transfected with either Flag-*DDX39B* WT or phosphomimetic (T344D or T389D) mutant plasmid. The *FOXP3* mRNA levels were normalized to *GAPDH* mRNA levels. Results (mean ± SEM, *n* = 3) are presented relative to those of vector controls.

**Figure 6:**
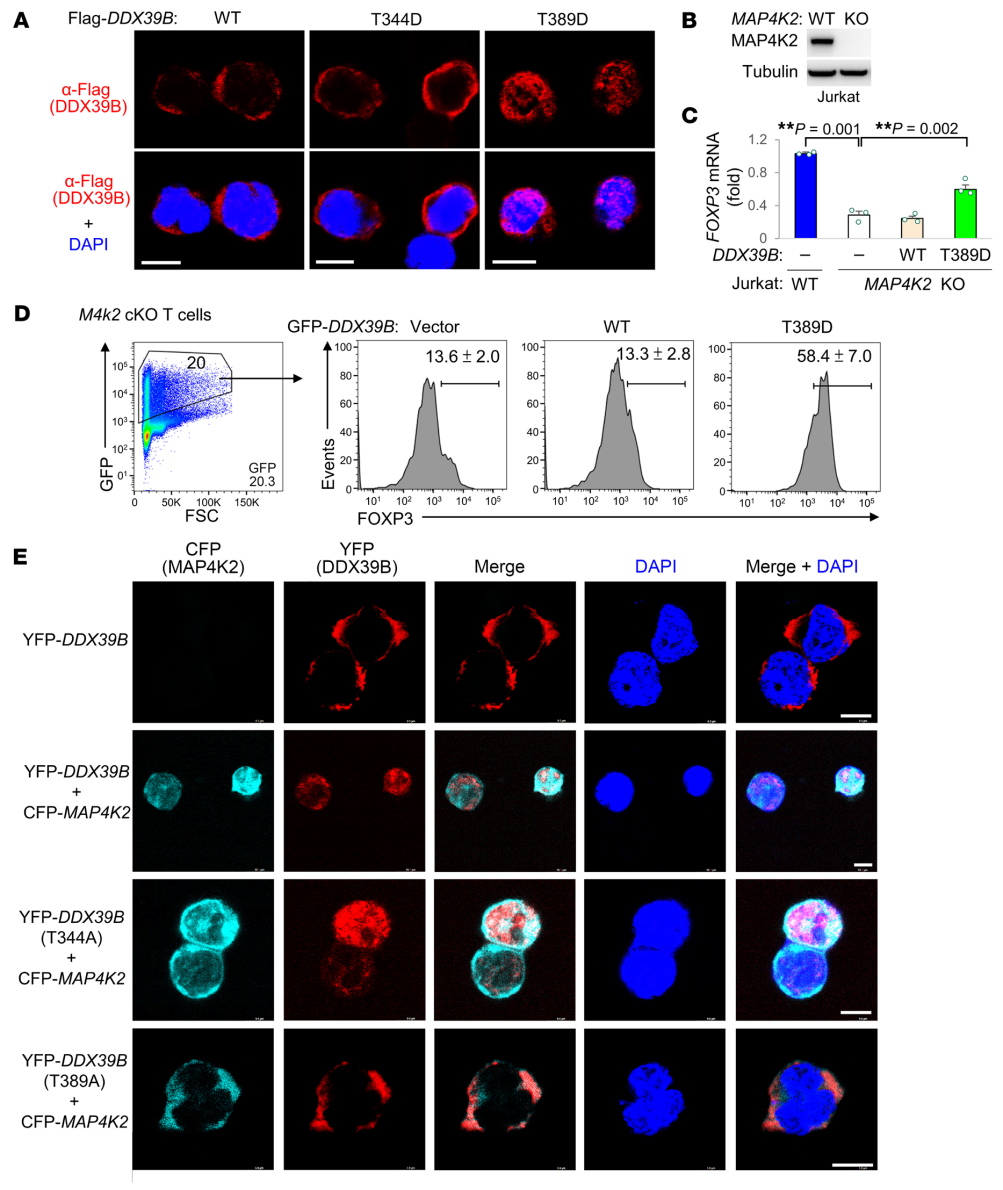
DDX39B Thr389 phosphorylation promotes its nuclear translocation. (**A**) Confocal microscopy of either Flag-tagged DDX39B WT or phosphomimetic (T344D or T389D) mutant proteins in the transfected Jurkat T cells. Nuclei were stained with DAPI. Scale bars: 10 μm. (**B**) Deficiency of MAP4K2 proteins in *MAP4K2* knockout (KO) Jurkat T cells was confirmed by immunoblotting (**C**) Real-time PCR of *FOXP3* mRNA levels in WT or *MAP4K2* KO Jurkat T cells transfected with either Flag-*DDX39B* WT or phosphomimetic (T389D) mutant plasmid. The mRNA levels of *FOXP3* were normalized to *GAPDH* mRNA levels. Results (mean ± SEM, *n* = 3) are presented relative to those of vector controls. (**D**) Flow cytometry analyses of the rescue of FOXP3^+^ cells in T-*Map4k2* conditional knockout (*M4k2* cKO) T cells transfected with either GFP-*DDX39B* WT or phosphomimetic (T389D) mutant plasmid. *n* = 4. (**E**) Confocal microscopy of CFP-fused MAP4K2, YFP-fused DDX39B, and YFP-fused DDX39B phosphodeficient (T344A or T389A) mutant proteins, as well as DAPI in the transfected Jurkat T cells. Scale bars: 10 μm. **P < 0.01 (1-way ANOVA and Tukey’s post hoc test). Data shown (**A**–**E**) are representative of 3 independent experiments.

Notably, unlike WT MAP4K2, MAP4K2 kinase-dead mutant failed to induce *FOXP3* levels in Jurkat T cells (Figure 5A). This suggests that MAP4K2 may phosphorylate its interacting protein DDX39B. Notably, the identified peptides of DDX39B proteins in the MAP4K2-immunocomplexes (Figure 3A) contained 7 serine/threonine phosphorylation sites at Ser38, Ser137, Ser153, Thr344, Thr383, Thr389, and Ser392 residues (Supplemental Figure 8). In vitro kinase assays using purified proteins showed that MAP4K2 phosphorylates DDX39B at a threonine site, while MAP4K2 kinase-dead mutant did not (Figure 5B). To identify the MAP4K2-targeted phosphorylation residue on DDX39B, in vitro MAP4K2-phosphorylated DDX39B proteins were isolated and subjected to mass spectrometry analyses. Thr389 and Thr344 on DDX39B protein were identified as MAP4K2-phosphorylated sites (Figure 5C). Mimicking MAP4K2-phosphorylated DDX39B protein, phosphomimetic DDX39B (T389D) mutant, but not (T344D) mutant, induced *Foxp3* mRNA levels (Figure 5D). Interestingly, DDX39B phosphomimetic (T344D) mutant protein was mainly restricted to the cytoplasm (Figure 6A), whereas DDX39B phosphomimetic (T389D) mutant protein was restricted to the nucleus (Figure 6A). These results suggest that MAP4K2-induced DDX39B Thr389, but not Thr344, phosphorylation is responsible for DDX39B nuclear translocation and subsequent *FOXP3* induction. Moreover, the reduction of *FOXP3* mRNA levels and FOXP3 protein levels in MAP4K2-KO Jurkat T cells and *Map4k2*-cKO T cells, respectively, was reversed by overexpression of DDX39B phosphomimetic (T389D) mutant protein in the absence of TGF-β stimulation (Figure 6, B–D). Conversely, MAP4K2-induced DDX39B nuclear translocation was inhibited by overexpression of DDX39B phosphodeficient (T389A) mutant in the presence of MAP4K2 (Figure 6E). Collectively, MAP4K2 directly phosphorylates DDX39B at Thr389 residue and subsequently induces DDX39B nuclear translocation, leading to *FOXP3* mRNA splicing and Treg differentiation.

**Figure 7:**
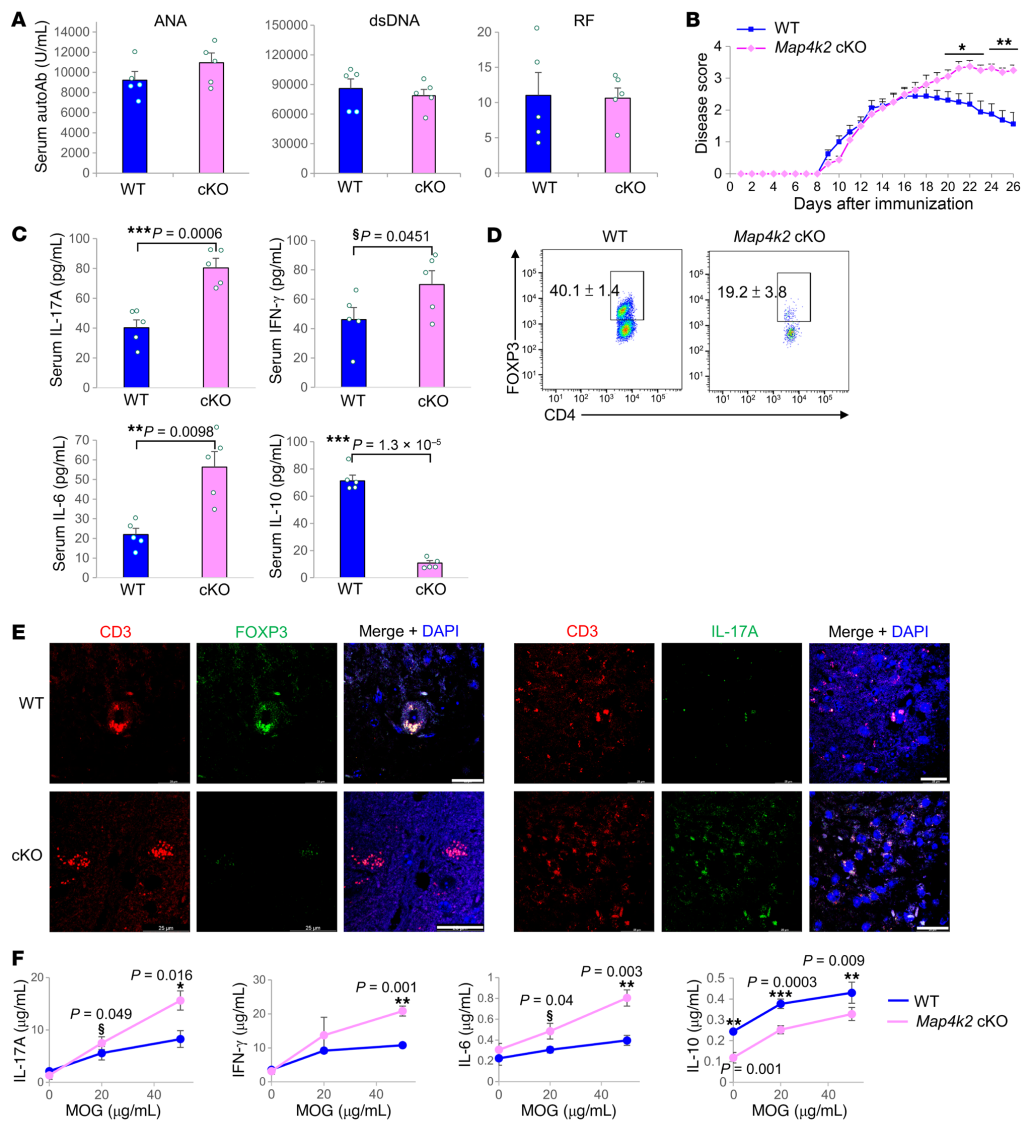
T cell–specific *Map4k2-*cKO mice display sustained induction of MOG-induced autoimmune encephalomyelitis. (**A**) ELISA of serum anti-ANA, anti-double strain DNA (dsDNA), and rheumatoid factor (RF) of 24-week-old T-*Map4k2* cKO (cKO) or WT (WT) mice. *n* = 5. (**B**–**F**) Induction of MOG-induced experimental autoimmune encephalomyelitis (EAE) using T-*Map4k2* cKO or WT mice. Clinical scores of mice are shown in mean ± SEM (**B**, *n* = 8). The cytokine levels in the sera of mice on day 26 of the MOG-induced EAE model were determined using ELISA assays (**C**, mean ± SEM, *n* = 5). Infiltrating Treg cells in the brain of diseased mice were determined by flow cytometry (**D**, *n* = 5). Confocal microscopy analyses of CD3 (red), FOXP3 (green, left panel), IL-17A (green, right panel), and DAPI in the brain tissues from diseased mice (**E**). Scale bars: 25 μm. MOG-specific T cell–mediated cytokine production was determined using ELISA assays (**F**, *n* =5). T cells were isolated from the lymph nodes of MOG-immunized mice on day 26 after immunization, followed by in vitro stimulation with MOG for 72 hours. ^§^*P* < 0.05; (1-tailed Student’s *t* test); *P < 0.05; ***P < 0.01; ***P < 0.001 (2-tailed Student’s *t* test). *M4k2* cKO, T cell–specific *Map4k2* conditional knockout (*Map4k2*^fl/fl^;*Cd4*-Cre); WT (*Map4k2*^fl/fl^). Data shown are representative of 3 independent experiments.

Treg quantity and function are defected in autoimmune disease (20). Notably, T-*Map4k2* cKO mice did not manifest spontaneous autoimmune symptoms and displayed normal levels of autoantibodies (Figure 7A). The induction of experimental autoimmune encephalomyelitis (EAE) is initiated by inflammatory Th1 and Th17 cells during the acute phase, and the EAE induction in the remission phase is attenuated by Treg cells. To study the role of MAP4K2 in Treg function in vivo, we performed the EAE model using T-*Map4k2* cKO mice. After myelin oligodendrocyte glycoprotein (MOG) immunization, the clinical score of T-*Map4k2* cKO mice during the acute phase was comparable with that of WT mice (Figure 7B). The clinical score of WT mice during the remission phase was declined as expected (Figure 7B), whereas the clinical score of T-*Map4k2* mice remained high during the remission phase (Figure 7B). The levels of proinflammatory cytokines IL-17A, IL-6, and IFN-γ were higher in the serum of T-*Map4k2* cKO mice than those of WT mice (Figure 7C). In contrast, the levels of anti-proinflammatory cytokine IL-10 were significantly decreased in T-*Map4k2* cKO mice (Figure 7C). T-*Map4k2* cKO mice also showed reduction of CNS-infiltrating Treg cells during the remission phase (Figure 7, D and E). The levels of infiltrating Th17 cells were increased, but the levels of Treg cells were decreased, in the brain tissue of T-*Map4k2* cKO mice compared with those of WT mice (Figure 7E). Moreover, in vitro MOG-restimulated IL-17A, IL-6, and IFN-γ production was increased in lymph node T cells isolated from MOG-immunized T-*Map4k2* cKO mice, whereas IL-10 production was decreased (Figure 7F). To validate the MAP4K2 function in Treg-mediated attenuation of autoimmunity, the EAE model was performed using Treg-specific *Map4k2*-deficient (*Map4k2*^fl/+^;*Foxp3*-Cre) mice (Supplemental Figure 9). The MOG-immunized Treg-specific *Map4k2*-deficient mice displayed similar phenotypes (Supplemental Figure 9) to those of T *Map4k2* cKO mice (Figure 7). These results suggest that MAP4K2 plays a critical role in Treg suppressive function in vivo.

**Figure 8:**
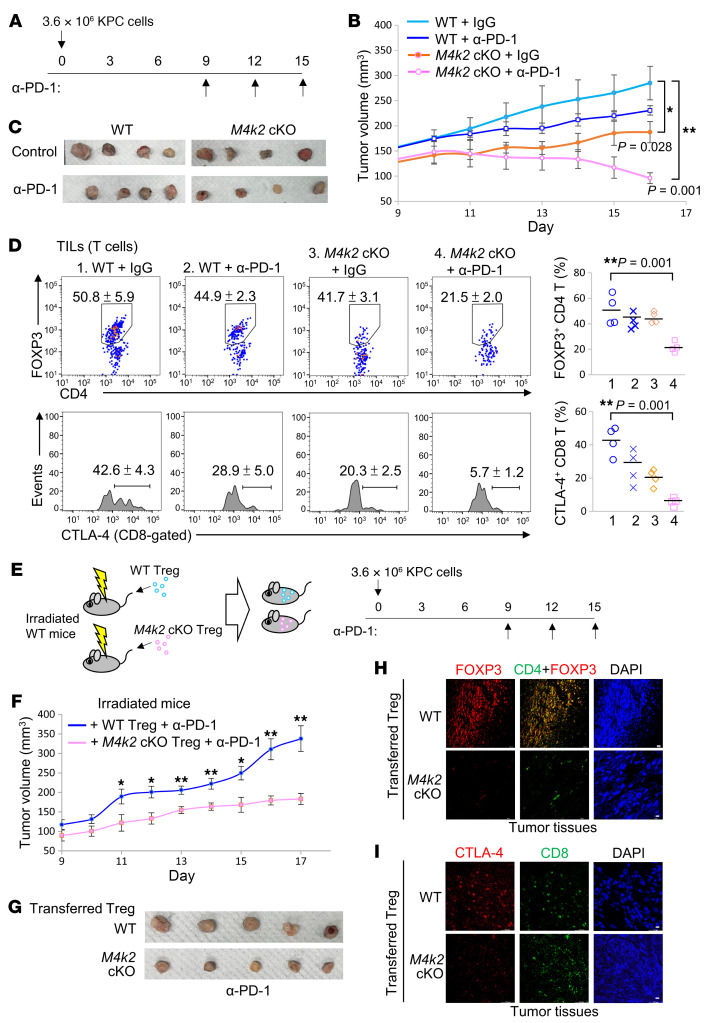
MAP4K2 deficiency in T cells strengthens cancer immunotherapy in mice. (**A**–**D**) Induction of syngeneic pancreatic cancer model in T-*Map4k2* (*M4k2*) cKO and WT mice by subcutaneous injection with KPC pancreatic cancer cells. Tumor-bearing mice were intraperitoneally injected with 200 μg anti–PD-1 antibody or control antibody (IgG) on days 9, 12, and 15 (**A**). *n* = 4 per group. Tumor volumes of the tumor tissues on the back of tumor-bearing T-*Map4k2* cKO or WT mice were measured from day 9 to day 16 (**B**). Tumor tissues were harvested from the backs of mice on day 16 (**C**). The tumor-infiltrating Treg (CD4^+^FOXP3^+^) cells and CD8 T cells of tumor tissues were determined by flow cytometry (**D**). (**E**–**I**) Induction of syngeneic KPC pancreatic cancer model in irradiated WT mice transferred with Treg cells from T-*Map4k2* (*M4k2*) cKO or WT mice. Tumor-bearing mice were intraperitoneally injected with 200 μg anti–PD-1 antibody on days 9, 12, and 15 (**E**). *n* = 5 per group. Tumor volumes of the tumor tissues on the back of tumor-bearing T-*Map4k2* cKO or WT mice were measured from day 9 to day 17 (**F**). Tumor tissues were harvested from the back of mice on day 17 (**G**). The tumor-infiltrating Treg (CD4^+^FOXP3^+^) cells and exhausted (CTLA-4^+^) CD8^+^ T cells of tumor tissues were detected by confocal microscopy (**H** and **I**). For (**B** and **D**), *P < 0.05; **P < 0.01 (1-way ANOVA and Tukey’s post hoc test); for (**F**), *P < 0.05; **P < 0.01 (2-tailed Student’s *t* test).

Treg cells also play a critical role in the suppression of anti-tumor immunity. We next studied whether MAP4K2 regulates Treg-mediated antitumor immunity using syngeneic KPC pancreatic cancer model. After subcutaneous injection with KPC pancreatic cancer cells for 9 days, the tumor tissues on the back of tumor-bearing mice were evident in both WT and T-*Map4k2* cKO mice (Figure 8, A and B). The tumor-bearing mice were then injected with anti–PD-1 antibody or IgG control antibody every 3 days. The tumor volumes were slightly decreased in anti–PD-1–treated WT mice compared with the control antibody group (Figure 8C). Interestingly, tumor volumes were significantly decreased in T-*Map4k2* cKO mice compared with those of WT mice (Figure 8C). Moreover, the infiltrating Treg population was drastically reduced in the tumor tissues of T-*Map4k2* cKO mice treated with either control antibody or anti–PD-1 antibody (Figure 8D). The percentages of CTLA-4^+^ CD8^+^ (exhausted) (21, 22) cytotoxic T cells were also decreased in the tumor tissues of T-*Map4k2* cKO mice (Figure 8D). Furthermore, IHC assays showed that DDX39B proteins were mainly localized in the nucleus of FOXP3^+^ T cells in the tumor tissue of WT mice, whereas DDX39B proteins were mainly localized in the cytoplasm of *Map4k2* cKO T cells (Supplemental Figure 10). The data support that MAP4K2 induces DDX39B nuclear translocation and subsequent FOXP3 expression in tumor-infiltrating Treg cells.

To study the intrinsic role of MAP4K2 in Treg-mediated antitumor immunity, Treg cells from T- *Map4k2* cKO or WT mice were adoptively transferred into the irradiated recipient mice, followed by the injection of KPC tumor cells to the back of the recipient mice. The tumor-bearing mice were injected with anti–PD-1 antibody every 3 days (Figure 8E). The recipient mice transferred with T-*Map4k2* cKO Treg cells displayed a reduction of tumor volumes compared with those of recipient mice transferred with WT Treg cells (Figure 8, F and G). The recipient mice transferred with T-*Map4k2* cKO Treg cells also displayed a decrease of the tumor-infiltrating Treg cells and exhausted (CTLA-4^+^) CD8^+^ T cells (Figure 8, H and I).

**Figure 9:**
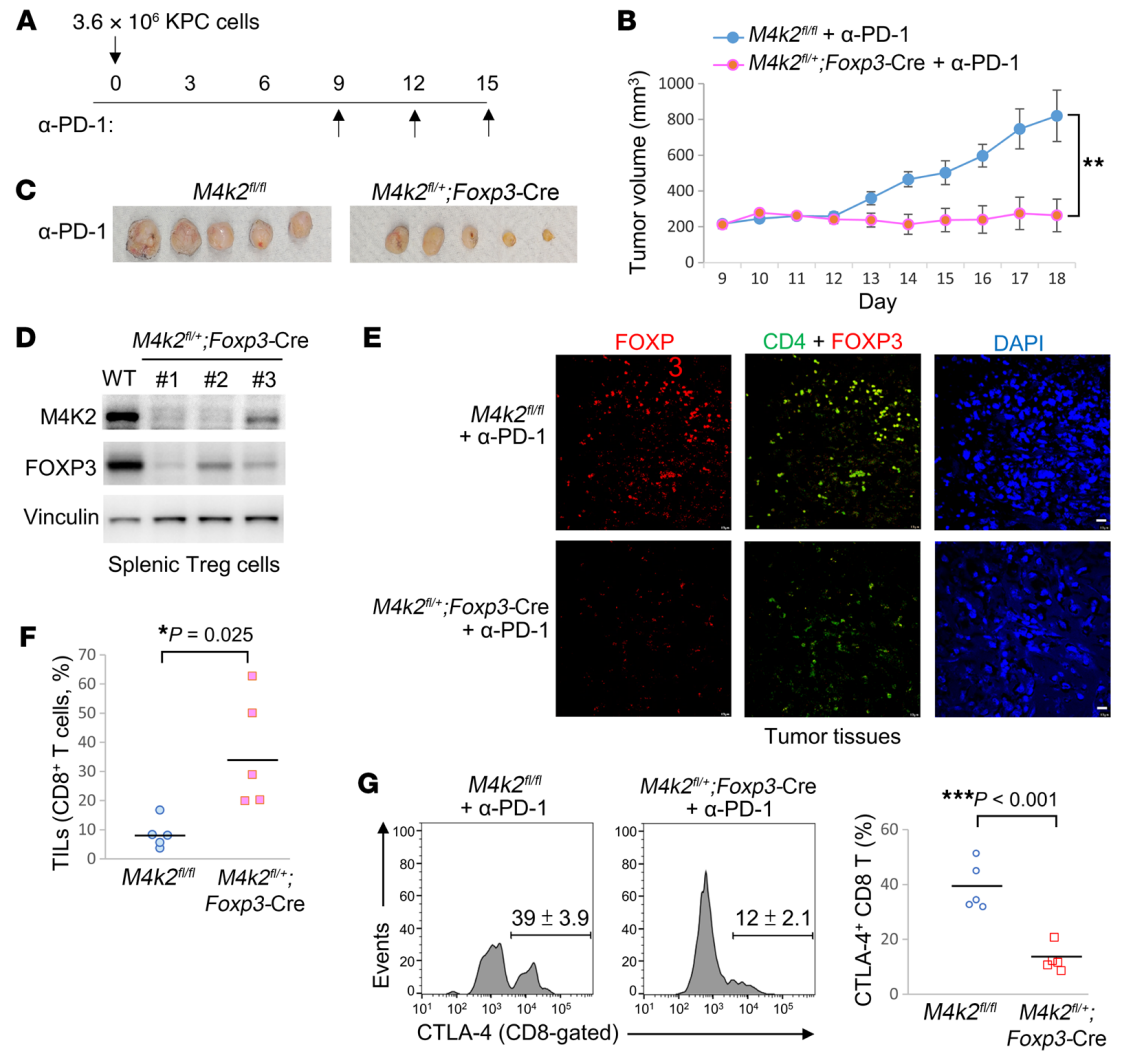
MAP4K2 suppresses Treg-mediated antitumor immunity in mice. (**A**–**G**) Induction of syngeneic pancreatic cancer model in Treg-specific *Map4k2*-deficient mice and WT mice by subcutaneous injection with KPC pancreatic cancer cells. Tumor-bearing mice were intraperitoneally injected with 200 μg anti–PD-1 antibody on day 9, 12, and 15 (**A**). Tumor volumes of the tumor tissues on the back of tumor-bearing Treg-specific *Map4k2-*deficient (*M4k2*-deficient) mice or WT mice were measured from day 9 to day 18 (**B**). *n* = 5 per group. Tumor tissues were harvested from the back of mice on day 18 (**C**). The protein levels of MAP4K2, FOXP3, and vinculin of splenic CD4^+^CD25^+^ T cells were determined by immunoblotting analyses (**D**). Confocal images showed CD4 (green), FOXP3 (Red), and DAPI of tumor-infiltrating Treg cells (**E**). Scale bars: 10 μm. Tumor-infiltrating CD8^+^ T cells (**F**) and CTLA-4–positive CD8^+^ T cells (**G**) of tumor tissues were determined by flow cytometry. WT (*Map4k2*^fl/fl^); *M4k2*^fl/+^;*Foxp3*-Cre, Treg-specific *Map4k2*-deficient mice. *P < 0.05; **P < 0.01; ***P < 0.001 (2-tailed Student’s *t* test).

To further validate the intrinsic role of MAP4K2 in Treg-mediated antitumor immunity, we also performed syngeneic KPC pancreatic cancer model using Treg-specific *Map4k2*-deficient (*Map4k2*^fl/+^;*Foxp3*-Cre) mice (Figure 9, A–G). The tumor-bearing mice were injected with anti–PD-1 antibody every 3 days. The tumor volumes were significantly decreased in *Map4k2*^fl/+^;*Foxp3*-Cre mice compared with those of WT (*Map4k2*^fl/fl^) mice (Figure 9, B and C). The MAP4K2 deficiency in splenic CD4^+^CD25^+^ T cells of *Map4k2*^fl/+^;*Foxp3*-Cre mice was confirmed by immunoblotting analysis (Figure 9D), and FOXP3 levels were concomitantly decreased (Figure 9D). The tumor-infiltrating Treg cells were decreased in *Map4k2*^fl/+^;*Foxp3*-Cre mice (Figure 9E); in contrast, the tumor-infiltrating CD8^+^ T cells were increased in *Map4k2*^fl/+^;*Foxp3*-Cre mice (Figure 9F). Moreover, the percentages of CTLA-4^+^ — an exhaustion marker (22) — cells were decreased in the tumor-infiltrating CD8^+^ T cells of tumor-bearing *Map4k2*^fl/+^;*Foxp3*-Cre mice (Figure 9G). Collectively, these results suggest that MAP4K2 deficiency results in reduction of Treg population and induction of cytotoxic T cell population, contributing to the enhancement of anticancer immunity.

**Figure 10:**
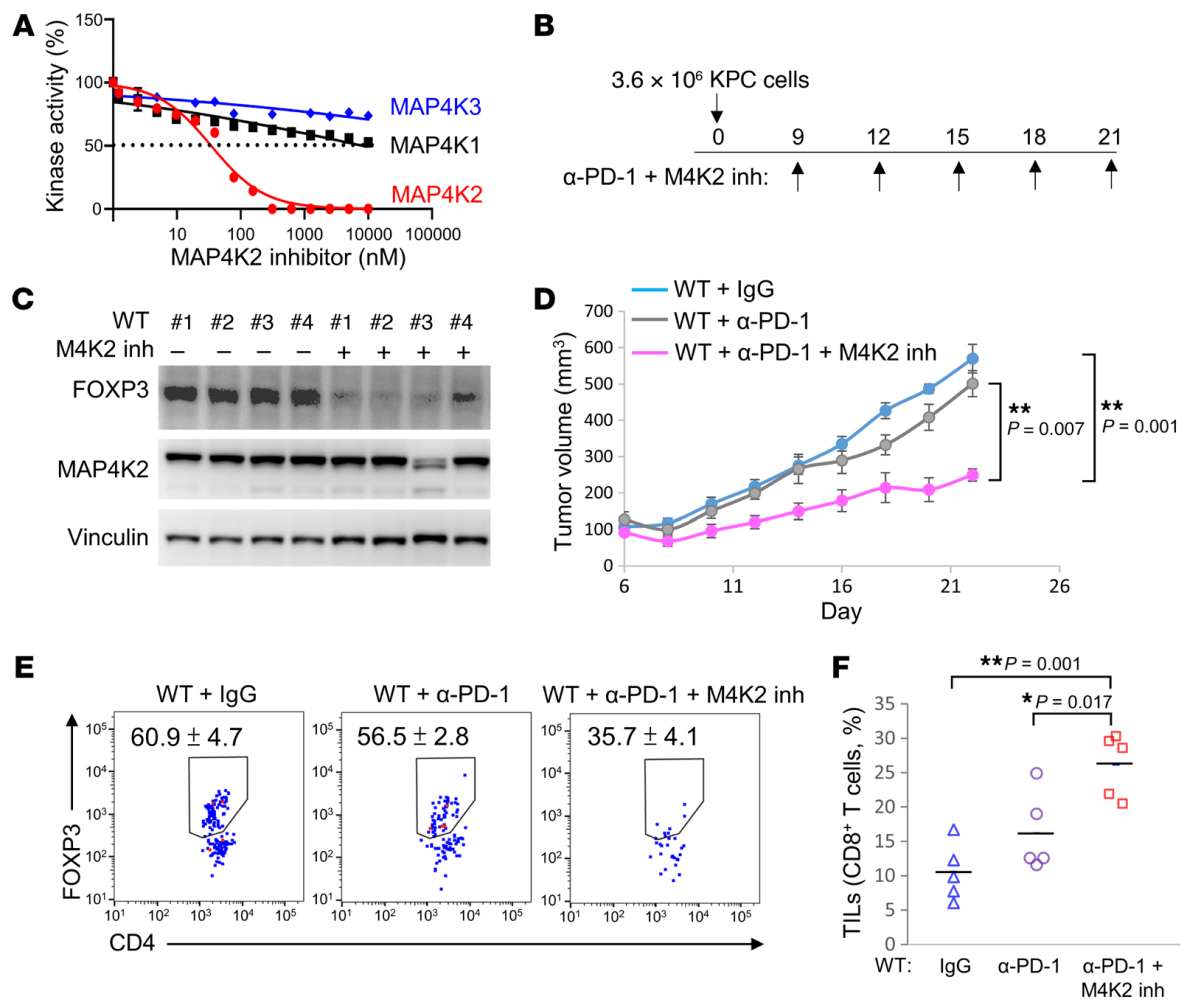
Syngeneic pancreatic cancer is inhibited by MAP4K2 inhibitor treatment in mice. (**A**) In vitro kinase assays using 50 ng each of purified MAP4K1 (HPK1), MAP4K2, and MAP4K3 proteins plus different concentrations of the MAP4K2 inhibitor TL4-12. IC_50_ of TL4-12 to MAP4K2 was 34.49 nM. (**B**–**F**) Induction of syngeneic pancreatic cancer model in TL4-12–treated mice by subcutaneous injection with KPC pancreatic cancer cells. Tumor-bearing mice were intraperitoneally injected with 200 μg anti–PD-1 antibody and the MAP4K2 inhibitor (M4K2 inh, TL4-12) on days 9, 12, 15, 18, and 21 (**B**). *n* = 4 per group. FOXP3 and MAP4K2 protein levels in the spleens of mice were determined by immunoblotting (**C**). Tumor volumes of the tumor tissues on the back of mice were measured from day 9 to day 22 (**D**). Infiltrating Treg (CD4^+^FOXP3^+^) cells of tumor tissues were determined by flow cytometry (**E**). Tumor-infiltrating CD8^+^ T cells of tumor tissues were determined by flow cytometry (**F**), *n* = 5, means ± SEM are shown. *P < 0.05; **P < 0.01 (1-way ANOVA and Tukey’s post hoc test). Data shown (**A** and **C**–**F**) are representative of 3 independent experiments.

To evaluate whether MAP4K2 is a druggable target for cancer immunotherapy, the MAP4K2 kinase inhibitor TL4-12 (12) was administered to KPC tumor–bearing WT mice (Figure 10, A and B). The MAP4K2 inhibitor TL4-12 selectively suppressed the kinase activity of MAP4K2 but not other related members-MAP4K1 or MAP4K3 (Figure 10A). The FOXP3 levels were indeed decreased in the splenic T cells of mice treated with TL4-12 (M4K2 inh) (Figure 10C). The volumes of KPC tumor tissues in WT mice were very slightly decreased by anti–PD-1 treatment (Figure 10D). Consistent with the result of T-*Map4k2* cKO mice, tumor volumes of anti–PD-1–treated WT mice were significantly decreased by TL4-12 treatment (Figure 10D). Moreover, the infiltrating Treg cells were decreased in the tumor tissues of TL4-12–treated mice compared with those of untreated (anti–PD-1 alone) mice (Figure 10E). The tumor-infiltrating CD8^+^ T cells were significantly increased in the tumor tissues of TL4-12–treated mice (Figure 10F). The data suggest that the MAP4K2 inhibitor efficiently suppresses Treg population in vivo, and MAP4K2 inhibitors may be potential therapeutics for cancer combination immunotherapy.

**Figure 11:**
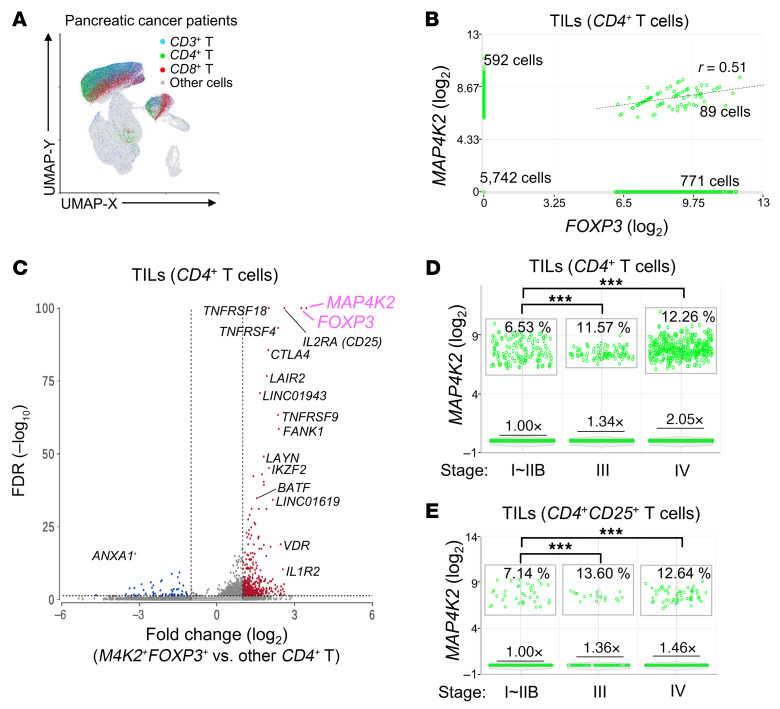
*MAP4K2* transcripts are increased in infiltrating Treg cells of human pancreatic cancer patients. (**A**–**E**) scRNA-seq data showed the distribution of *CD3*^+^ T cells (blue), *CD3*^+^*CD4*^+^ cells (green), *CD3*^+^*CD8*^+^ cells (red), and other cells (gray) from pancreatic cancer tissues of 27 patients (**A**). Data are presented in Uniform Manifold Approximation and Projection (UMAP). *MAP4K2* mRNA levels were correlated with *FOXP3* mRNA levels in the tumor-infiltrating *MAP4K2*^+^*FOXP3*^+^ T cells of 27 patients with pancreatic cancer (**B**). The volcano plot of the selected differentially expressed genes in the tumor-infiltrating *MAP4K2*^+^*FOXP3*^+^*CD4*^+^ T cells and other tumor-infiltrating *CD4*^+^ T cells. FDR, false discovery rate (**C**). Plots show *MAP4K2* mRNA levels of the tumor-infiltrating *CD4*^+^ T cells or *CD4*^+^*CD25*^+^ T cells from 20 untreated patients with different stages of pancreatic cancer (**D** and **E**). The folds were the least square means normalized to the least square mean of cells from resectable patients. The percentages of *MAP4K2*^+^ cells were shown in rectangles. ***P < 0.001 (Kruskal-Wallis test).

Our results derived from cells and mice showed that MAP4K2 is a key kinase controlling Treg differentiation and is a potential target for cancer immunotherapy. Treg cells are abundant in the tumor tissue of cancer patients (23). To study whether MAP4K2 levels are associated with Treg population in human tumor tissues, we performed data mining of a published scRNA-seq dataset of human pancreatic cancer patients (24). The cells of the tumor tissues from 27 patients with pancreatic cancer were analyzed by scRNA-seq (24). The UMAP plot showed 36,891 tumor-infiltrating T cells among 148,335 cells (Figure 11A). Interestingly, 89 of 7,194 CD4 T cells in the tumor tissues from 27 patients with pancreatic cancer were *MAP4K2* and *FOXP3* double-positive (*MAP4K2^+^FOXP3*^+^) *CD4*^+^ T cells, which showed a correlation (linear regression, r = 0.51) between *MAP4K2* and *FOXP3* levels (Figure 11B). The tumor-infiltrating *MAP4K2*^+^*FOXP3*^+^ CD4^+^ T cells displayed a drastic induction of *CD25*, *CTLA4, TNFRSF18, TNFRSF4*, and *BATF* compared with other *CD4*^+^ T cells (Figure 11C). Moreover, *MAP4K2* mRNA levels of the tumor-infiltrating *CD4*^+^ T cells or Treg (*CD4^+^CD25*^+^) cells were significantly increased in patients with cancer metastasis or with locally advanced cancer compared with resectable cancer (Figure 11, D and E). The frequencies of *MAP4K2*^+^ cells in the tumor-infiltrating *CD4*^+^ T cells or Treg (*CD4*^+^*CD25*^+^) cells were also increased in patients with cancer metastasis or with locally advanced cancer (Figure 11, D and E). In addition, *MAP4K2* levels of the tumor-infiltrating *CD4*^+^ T cells were decreased in patients treated with chemotherapy (Supplemental Figure 11A). Consistent with the data of the original publication (24), the levels of the exhaustion markers *CTLA-4* and *TIGIT* in the tumor-infiltrating *CD8*^+^ T cells were decreased in patients with pancreatic cancer treated with chemotherapy (Supplemental Figure 11, B and C). The data suggest that MAP4K2 promotes Foxp3 expression and Treg differentiation, contributing to cancer progression and tumor immunity.

## Discussion

A key finding of this study was the identification of a Treg regulator, MAP4K2. MAP4K2 promotes DDX39B-mediated *FOXP3* pre-mRNA splicing to mature *FOXP3* mRNA in T cells, leading to the differentiation of Treg cells (Figures 1–6). Consistently, data mining of Human Protein Atlas (HPA) database showed that MAP4K2 transcript level in Treg subset is the highest among individual T cell subsets. Our data also showed that MAP4K2 protein levels were induced in Treg cells compared with other T cell subsets. Conversely, *Map4k2* deficiency in T cells resulted in the increase of *Foxp3* pre-mRNA levels, decrease of *Foxp3* mRNA levels, and subsequent downregulation of Treg differentiation. Notably, T-*Map4k2* cKO mice did not manifest spontaneous autoimmune symptoms (Figure 7). In addition, antipancreatic cancer immunotherapy by anti–PD-1 antibody was drastically strengthened in T-*Map4k2* cKO mice (Figure 8) or Treg-specific *Map4k2*-deficient mice (Figure 9). Pharmacological inhibition of MAP4K2 in mouse cancer models showed similar results (Figure 10). Interestingly, *Map4k2* deficiency in Treg cells of tumor-bearing mice contributed to the induction and activation of tumor-infiltrating CD8^+^ T cells. Consistently, scRNA-seq data of human pancreatic cancer patients showed that *MAP4K2* levels in the tumor-infiltrating Treg cells were increased in patients with the late-stage pancreatic cancer (Figure 11E). Furthermore, scRNA-seq data of human pancreatic cancer patients showed a correlation between *MAP4K2* and *FOXP3* levels in *MAP4K2* and *FOXP3* double-positive CD4^+^ T cells (Figure 11B). These results suggest that MAP4K2 is a key activator of FOXP3 expression and Treg differentiation, leading to the attenuation of anti-cancer immunity. The data support that MAP4K2 is a biomarker and an immunotherapeutic target for cancer.

One exciting finding in this report is that MAP4K2 directly phosphorylates the DEAD box protein DDX39B at Thr389 residue, resulting in nuclear translocation of the phosphorylated DDX39B and subsequent induction of mature *Foxp3* mRNA. The MAP4K2 activity and the MAP4K2-DDX39B interaction were induced by TGF-β stimulation in T cells. Notably, the MAP4K2-DDX39B interaction occurred in transfected HEK293T cells without TGF-β stimulation (Figure 3B), and the MAP4K2-DDX39B interaction was unaffected by the loss of MAP4K2 kinase activity (Supplemental Figure 7B). These results suggest that MAP4K2 kinase activity is not required for MAP4K2-DDX39B interaction. It remains possible that TGF-β signaling induces additional modification on DDX39B protein that regulates the MAP4K2-DDX39B interaction; however, this is beyond the scope of this study. DDX39B shuttles between the nucleus and cytoplasm (25); however, the mechanism of DDX39B nuclear translocation is unclear. The nuclear translocation of human DDX39B protein is independent of its putative NLS domains and its interacting pore-associated protein Rae1 (25). In this report, our findings showed that MAP4K2 directly induced DDX39B Thr389 phosphorylation and promoted DDX39B nuclear translocation. Also, MAP4K2-phosphorylated DDX39B could reciprocally facilitate the nuclear translocation of MAP4K2. In support of this possibility, DDX39B promotes nuclear translocation of PKM2 in colorectal cancer cells (26), contributing to the induction of oncogenes and glycolysis-associated genes (26). It would be interesting in the future to study whether MAP4K2, after translocation to the nucleus, could independently, or cooperatively with DDX39B, regulate other splicing factors in spliceosomes.

DDX39B and DDX39A proteins show a very high sequence identity (> 90%), and the functional redundancy of DDX39B and DDX39A in RNA processing of several target genes has been reported (27). In contrast, the role of DDX39B in *FOXP3* RNA splicing is unique, and DDX39B deficiency in regulating *FOXP3* RNA splicing could not be compensated by DDX39A (27). Consistently, our data showed that *FOXP3* mRNA levels were abolished in *DDX39B*-KO Jurkat T cells. These findings indicate that DDX39B is an indispensable activator for *FOXP3* RNA splicing.

Taken together, the MAP4K2-DDX39B axis is critical in promoting *FOXP3* RNA splicing and inducing Treg differentiation, leading to the reduction of anticancer immunity. Conversely, MAP4K2 deficiency in vivo results in the enhancement of anticancer immunotherapy efficacy. Thus, inhibition of MAP4K2 levels or activity could be a useful therapeutic approach to improve cancer immunotherapy.

## Methods

### Sex as a biological variable.

Sex was not considered as a biological variable in the experiments using human peripheral blood T cells and the syngeneic cancer mouse model, but only female mice were examined for the EAE model because the disease modeled is only relevant in females.

### Mice.

All animal experiments were performed in the AAALAC-accredited animal housing facilities according to the protocols and guidelines (NHRI-IACUC-112052) approved by the Institutional Animal Care and Use Committee of National Health Research Institutes (NHRI). A C57BL/6N mouse embryonic stem cell clone (ID 113421, clone #CSD118574) with *Map4k2* KO-first allele from the Knockout Mouse Project (KOMP) was injected into C57BL/6J blastocysts to generate chimeric mice by NHRI Transgenic Mouse Core. The KO-first allele is initially at a nonexpressing state, but can be converted to a conditional floxed allele via Flp recombination using *Actin-Flp* transgenic mice (JAX #003800). Floxed *Map4k2* mice were then backcrossed with wild-type C57BL/6J mice for 10 generations. T cell–specific *Map4k2* conditional KO (T-*Map4k2* cKO) mice were generated by crossing floxed *Map4k2* mice and *Cd4*-Cre transgenic mice (JAX #022071). For Treg-specific *Map4k2* deficient (*Map4k2*^fl/+^;*Foxp3*-Cre) mice, *Map4k2*^fl/fl^ mice were crossed with *Foxp3*-Cre transgenic mice to generate *Map4k2*^fl/+^;*Foxp3*-Cre offspring (41%) mice. Notably, B6129S *Foxp3*-Cre transgenic mice (JAX #023161) have been backcrossed with C57BL/6J WT mice for 5 generations (about 97% C57BL/6J background) in our laboratory. The frequencies of *Map4k2*^fl/fl^;*Foxp3*-Cre mice (in C57BL/6J background) were very low (14 of 186, 7.5%) after crossing *Map4k2*^fl/fl^ mice with *Map4k2*^fl/+^;*Foxp3*-Cre mice. Thus, it is difficult to obtain enough sex- and age-matched *Map4k2*^fl/fl^;*Foxp3*-Cre mice for mouse disease models. Alternatively, we used *Map4k2*^fl/+^;*Foxp3*-Cre mice, which displayed comparable Treg deficiency and similar effects to those of T cell–specific *Map4k2* conditional knockout (T-*Map4k2* cKO) mice. The mice were maintained in temperature-controlled and pathogen-free cages.

### Human participants.

This study was conducted in accordance with the Helsinki Declaration. Three healthy individuals were enrolled in this study; these healthy volunteers were referred to National Taiwan University Hsin-Chu Hospital in Taiwan. Written informed consents (approved by the Institutional Review Board at National Taiwan University Hsin-Chu Hospital, Taiwan [#109-008-E] were obtained from individual volunteers before enrollment in this study. The Institutional Review Boards at National Taiwan University Hsin-Chu Hospital, Taiwan [#109-008-E] and National Health Research Institutes, Taiwan [EC-1060701-E] approved the experiments.

The scRNA-seq dataset of tumor tissues from 27 patients with pancreatic ductal adenocarcinoma were retrieved from NCBI SRA Bio project #PRJNA843078 (24). The scRNA-seq data of pancreatic cancer were analyzed by Partek Flow software.

### scRNA-seq.

Splenic and lymph-node T cells of T-*Map4k2* cKO and WT mice were purified by negative selection using magnetic cell separation columns (Miltenyi Biotec). CD4^+^ T cells and CD8^+^ T cells were labelled by the AbSeq anti-CD4 and anti-CD8 antibody-oligonucleotide conjugates (BD Biosciences), respectively. The T cells were processed by BD Rhapsody Single-Cell Analysis System (BD Biosciences). The scRNA-seq data of mice were analyzed by BD SEQGEQ (BD Biosciences) software, as well as the R package Seurat, as described previously (28–31). The scRNA-seq data of T-*Map4k2* cKO mice have been uploaded previously to a public, open-access repository (NCBI SRA #PRJNA990957). The data of WT mice and T-*Map4k2* cKO mice were SampleTag01 and SampleTag02, respectively. Of note, the data of WT mice and UBR2-KO mice were SampleTag03 and SampleTag04, respectively (32).

### EAE model.

Eight-week-old female mice were used for the induction of EAE model as described previously (8, 33). Mice were subcutaneously injected with myelin oligodendrocyte peptide (MOG, 200 μg/mouse) in complete Freund’s adjuvant (containing 250 μg *Mycobacterium tuberculosis* (Difco)) at 2 sites on the back. The immunized mice were also injected with pertussis toxin (Sigma-Aldrich, 200 ng/mouse) on days 0, 1, and 2. Clinical scores (8) of mice were daily monitored on a scale of 0–5 for 26 days. To collect the infiltrating immune cells from the brain and spinal cord of MOG-immunized mice, the brain and spinal cord were homogenized by gentleMACS Dissociator (Miltenyi Biotec), followed by incubation with RPMI media containing collagenase II (Sigma-Aldrich) at 37 °C for 15 minutes. The infiltrating immune cells were isolated by Percoll (Cytiva) gradient centrifugation and subjected to flow cytometry analysis. For MOG-specific T cell responses, 6 × 10^6^ cells were isolated from draining lymph nodes of individual mice on day 26 postimmunization and cultured in RPMI media containing MOG (0, 20, or 50 μg/mL) for 72 hours.

### Syngeneic KPC pancreatic cancer model and adoptive transfer experiments.

KPC pancreatic cancer cells were derived from C57B/6 KPC (*Kras*^LSL-G12D^, *Trp53*^R172H^, and *PDX-1*-Cre) mice (34). KPC pancreatic cancer cells (3.6 × 10^6^) were subcutaneously injected into the back of WT C57B/6J, *Map4k2*^fl/fl^, or *Map4k2*^fl/fl^;*Cd4*-Cre, or *Map4k2*^fl/+^;*Foxp3*-Cre mice to develop experimental tumors. Once the tumor tissues were evident on the back of mice, the tumor-bearing mice were then intraperitoneally injected with 200 μg anti–PD-1 antibody (BioLegend, #135250, Lot# B414816) or control antibody (BioLegend, #400566, Lot# B430990) every 3 days. For Figure 10, mice are also injected with the MAP4K2 inhibitor TL4-12 (1 μM, 100 μL/mouse). Mice were monitored for tumor formation, and the tumor size were measured with a caliper. The tumor volumes were calculated using the following formula (35): *V* = (*W^2^* × *L*) / 2. For the adoptive transfer experiment, CD4^+^CD25^+^ T cells were isolated from the spleen and lymph nodes of 5 donor mice (*Map4k2*^fl/fl^ or *Map4k2*^fl/fl^;*Cd4*-Cre mice) and then mixed with WT splenic CD8^+^ cells (1 × 10^7^ cells per recipient mouse). The cells were intravenously transferred into 5 irradiated (5.5 Gy; RS2000 irradiator, Rad Source) recipient mice. One day later, the recipient mice were subjected to KPC cancer cell injection.

### Cell lines and transfection.

Human Jurkat T leukemia (ATCC, TIB-152) and CRISPR & TARGATT gene editing Jurkat (Jurkat-Cas9, Applied StemCell) cell lines were cultured in RPMI 1640 medium containing 10% FBS, 100 units/mL penicillin, and 100 μg/mL streptomycin. *MAP4K2* knockout Jurkat T cells were generated by the CRISPR-Cas9 approach using Jurkat-Cas9 cells, which were transfected with the pGuide-EF1a-GFP plasmid (OriGene) containing the guide sequence 5′-aaagttcggacgtgaccgtgtcgc-3′. The human embryonic kidney HEK293T cell line (CRL-11268; ATCC) was maintained in DMEM medium containing 10% FBS, 100 units/mL penicillin, and 100 μg/mL streptomycin. All cells were verified to be negative for mycoplasma. Plasmids were transfected into HEK293T cells using polyethylenimine reagents (Sigma). Plasmids were transfected into Jurkat T cells using the ExTransfection Electroporation System (NanoEnteck) on 1,430 V for a duration of 30 ms and 1 pulse. For electroporation using primary T cells, the condition was 2,200 V, 30 ms duration, and 1 pulse.

### Plasmids.

The plasmid encoding 3xMyc-tagged human MAP4K2 protein was generated by cloning the *MAP4K2* cDNA into the vector pCMV-3Tag-9 (Agilent Technologies). The plasmid expressing MAP4K2 (K45A) kinase-dead mutant protein was generated from the Myc-*MAP4K2* plasmid by mutating lysine-45 to alanine using PCR-based site-directed mutagenesis. The plasmid expressing 3xFlag-tagged human DDX39B protein was generated by subcloning *DDX39B* cDNA into the vector pCMV6-AN-3DDK (OriGene). The plasmids expressing CFP-fused MAP4K2 and YFP-fused DDX39B proteins were constructed by subcloning the *MAP4K2* cDNA and *DDX39B* cDNA from the 3xMyc-*MAP4K2* plasmid and 3xFlag-*DDX39B* plasmid into the pCMV6-AC-CFP vector (OriGene) and pCMV6-AN-YFP vector (OriGene), respectively. The plasmids encoding DDX39B (T344A), (T389A), (T344D), and (T389D) mutant proteins were generated by mutating the indicated amino acid residues in either the 3xFlag-*DDX39B* or YFP-*DDX39B* plasmid.

### Reagents and antibodies.

The in-house–made rabbit antibody recognizing both human and murine MAP4K2 proteins was generated by immunization of a rabbit with MAP4K2 peptides (murine MAP4K2 epitope: ^232^SFQPPKLRDKTRWTQNFHH^250^; corresponding to the human MAP4K2 protein sequences ^230^SFQPPKLRDKTRWTQNFHH^248^). The in-house–made rabbit antibody recognizing both human and murine DDX39B proteins was generated by immunization of a rabbit with DDX39B peptides (human DDX39B epitope: ^18^VETAAGGDGAE^28^; corresponding to the murine DDX39B protein sequences ^18^VETAAGADGTE^28^). Anti-Myc (clone 9E10, #MABE282) and anti-Flag (clone M2, #F3165) monoclonal antibodies were purchased from Merck. AF594-conjugated anti-mouse IgG antibody (#ab150108) was purchased from Abcam. Anti-mCD3-RB705 (clone 145-2C11, #570560) antibody was purchased from BD Horizon. Anti-hCD3 (clone OKT3) were purified from mouse ascites by protein A-Sepharose chromatography. Anti-mCD4-pacific blue (clone RM4-5, #558107) and anti-mCD8-APC-Cy7 (clone 53-6.7, #557654) antibodies were purchased from BD Biosciences. Anti-FOXP3-PE (clone 150D, #320008), anti-mIL-4-PE (clone 11B11, #504104), anti-mIFN-γ-FITC (clone XMG1.2, #505806), and anti-CTLA4-APC (clone UC10-4B9, #106310) antibodies were purchased from BioLegend. Anti-mCD45-VioGreen antibody (clone REA737, #130-110-665) was purchased from Miltenyi Biotec. ELISA kits of mouse IFN-γ, IL-17A, IL-10, and IL-6 were purchased from BD Biosciences (#2760KI), BioLegend (#432505), R&D (#DY417), and Invitrogen (#88-7064-88), respectively.

### Determination of Foxp3 mRNA and pre-mRNA.

The determination and quantitation of mouse *Foxp3* mRNA and pre-mRNA were performed using methods described in a previous publication (19). The mouse *Foxp3* mRNA levels were determined by real-time PCR and normalized to mouse *Gapdh* levels. The *Foxp3* pre-mRNAs are the mRNAs lacking the splicing events across exon junctions. These *Foxp3* pre-mRNA levels were detected using primers targeting the individual junction regions of intron 2–exon 3, intron 4–exon 5, exon 6–intron 6, intron 7–exon 8, exon 9–intron 9, and intron 11–exon 12 by real-time PCR, followed by normalizing to mouse *Gapdh* levels. For calculating *Foxp3* pre-mRNA levels, the individual *Foxp3* pre-mRNA levels were further normalized to total *Foxp3* mRNA levels. For detection of mouse *Foxp3* mRNA and pre-mRNA levels by real-time PCR (19), the primer pairs for *Foxp3* mRNA: forward 5′-AGAGGCAGAGGACACTCAATG-3′, reverse 5′-TGGCGGGGTGGTTTCTGAAG-3′; *Foxp3* intron 2-exon 3: forward 5′-TATTAAGATGAGGCTCATGGCATC-3′, reverse 5′-CTGGAGAAGGGCCTGTAGG-3′; *Foxp3* intron 4-exon 5: forward 5′-GAGGGGCCTTCTGGAACAAG-3′, reverse 5′-AGCGTGGGAAGGTGCAGAG-3′; *Foxp3* exon 6-intron 6: forward 5′-TCGAGGAGCCAGAAGAGTTTC-3′, reverse 5′-GAGACGTGAAGCCTAGACAG-3′; *Foxp3* intron 7-exon 8: forward 5′-GCATGGGCAAAAGTGTGCC-3′, reverse 5′-CTCCCAGCTTCTCCTTTTCC-3′; *Foxp3* exon 9-intron 9: forward 5′-GGGAAGCCATGGCAATAGTTC-3′, reverse 5′-GGAGCTCCTTTGTACCCCC-3′; *Foxp3* intron 11-exon 12: forward 5′-GGCTAGACATGTGGGGAAG-3′, reverse 5′-ACTTGTGCAGGCTCAGGTTG-3′. For detection of human *Foxp3* mRNA by real-time PCR, the primer pairs were forward 5′-AGAAGCAGCGGACACTCAAT-3′ and reverse 5′-TGGCAGGATGGTTTCTGAAG-3′.

### T cell purification and in vitro T cell differentiation.

Primary murine CD4^+^ T cells were negatively selected from the spleen of mice using magnetically coupled antibodies against CD8 (clone 53-6.7, #100704, BioLegend), B220 (clone RA3-6B2, #553086, BD Biosciences), CD11b (clone M1/70, #553309, BD Pharmingen), CD11c (clone HL3, #553800, BD Biosciences), CD49b (clone DX5, #108904, BioLegend), and TER-119 (clone TER-119, #553672, BD Biosciences), as described previously (36). For the purification of CD4^+^CD25^+^ T cells, the purified CD4^+^ T cells were further positively selected using a biotin-conjugated antibody against CD25 (clone PC61, #102003, BioLegend) on a magnetic cell separation column (Miltenyi Biotec). T cell purification and in vitro Th1/Th2/Th17 differentiation using murine splenic T cells were performed using methods described previously (8, 33, 37, 38). For in vitro Treg differentiation, 5 × 10^5^ splenic CD4^+^CD25^+^ T cells were cultured for 5 days in 1 mL RPMI-1640 (Thermo Fisher Scientific) in 48-well plates coated with anti-CD3 (2.5 μg, clone 145-2C11, #553057, BD Biosciences) and anti-CD28 (2.5 μg, clone 37.51, #553294, BD Biosciences) antibodies. The RPMI media contained IL-2 recombinant proteins (10 ng/mL, #P04351, R&D Systems), TGF-β (1 or 2 ng/mL, #P01137, R&D Systems), anti-IL-4 (2.5 μg/mL, clone 11B11, #504122, BioLegend) antibody, and anti-IFN-γ (2.5 μg/mL, clone XMG1.2, #505834, BioLegend) antibody (38).

### In vitro Treg suppression assays.

For preparation of effector T cells, splenic T cells were isolated from WT mice, followed by depletion of regulatory T cells using biotin-conjugated anti-mouse CD25 antibody (clone PC61, #102003, BioLegend) by MACS magnetic-bead separation (Miltenyi Biotec). For determination of effector T cell proliferation, effector T cells were labeled with 1 μM CFSE (Thermo Fisher Scientific). Murine CD4^+^CD25^+^ T cells (1.66 × 10^5^ or 0.8 × 10^5^) from the spleen and lymph nodes of WT or T-*Map4k2* cKO mice were cocultured with the CFSE-labelled effector T cells (5 × 10^5^) for 4 days under the stimulation of anti-CD3 antibody (1 μg/mL, BD Biosciences) (8, 38).

### Immunofluorescence staining and confocal microscopy.

Jurkat T cells were fixed with cold methanol for 2 min, followed by permeabilization using Triton X-100 (0.1%) for overnight. The fixed T cells were incubated with anti-Flag monoclonal antibody (1:500, clone M2, #F3165, Merck) at 4°C for overnight, followed by incubation with AF594 anti-mouse IgG antibody (1:400, #ab150108, Abcam) for 1 hour. The cell nucleus was stained with DAPI (#DUO82040, Sigma-Aldrich). The images were acquired by Leica Stellaris 8 confocal microscope.

### Co-IP and FRET.

The interaction between MAP4K2 and DDX39B proteins was determined by co-IP and fluorescence resonance energy transfer (FRET; detecting protein-protein interaction <10 nm) (39), as described previously (30, 40).

### In situ proximity ligation assay (PLA) technology.

In situ PLA assays were performed using the Duolink In Situ-Red kit (Sigma-Aldrich), according to the manufacturer’s instructions described previously (41, 42). Briefly, in vitro differentiated Treg cells were incubated with anti-MAP4K2 (in-house made) and anti-DDX39B (in-house made) antibodies, followed by anti-rabbit or anti-mouse IgG antibodies conjugated with oligonucleotides (PLA probes, Sigma-Aldrich). After ligation and amplification reactions, PLA signals from each pair of PLA probes in close proximity (< 40 nm; between MAP4K2 and DDX39B) were visualized as individual red dots and analyzed by Leica TCS SP5 II microscope.

### In vitro MAP4K2 kinase assays.

To determine MAP4K2 kinase activity in Jurkat T cells (Supplemental Figure 7A), Flag-tagged MAP4K2 proteins were isolated from protein lysates of Jurkat T cells transfected with Flag-*MAP4K2* plasmid. The Flag-tagged MAP4K2 proteins were purified by peptide-elusion purification using Flag peptides. The kinase activity of Flag-tagged MAP4K2 was determined by measuring the rate of ADP production in the in vitro kinase reaction using ADP-Glo Kinase Assay kit (Promega, V6930) and myelin basic protein (MBP; Upstate) as the substrate. To determine MAP4K2-targeted phosphorylation sites on DDX39B protein, the purified DDX39B protein was used as the substrate for in vitro MAP4K2 kinase assays (Figure 5B). Myc-tagged MAP4K2, Myc-tagged MAP4K2 (K45A) kinase-dead mutant, and Flag-tagged DDX39B proteins were immunoprecipitated from lysates of HEK293T transfectants, followed by peptide-elusion purification using Myc or Flag peptides. Purified DDX39B proteins were incubated with purified MAP4K2 WT or kinase-dead (K45A) mutant proteins and ATP (0.1 mM) in the kinase buffer (40 mM Tris-HCl, pH7.5, 20 mM MgCl_2_, 0.1 mg/mL BSA, 10 mM DTT and 0.1 mM Na_3_VO_4_) at 37°C for 30 min. The reaction products were denatured in sample buffer at 95°C for 3 min, followed by immunoblotting with anti-pan-phospho-serine (Merck, clone 4A4, #05-1000) and anti-pan-phospho-threonine (Cell Signaling, clone 42H4, #9386) monoclonal antibodies.

### Liquid chromatography-mass spectrometry analysis.

To identify MAP4K2-interacting proteins, proteins of the MAP4K2 immunocomplexes were digested with trypsin and subjected to LC-MS/MS analyses by Orbitrap Fusion Tribrid mass spectrometer, as described previously (30, 40). For the identification of MAP4K2-phosphorylated residues on the DDX39B protein, Flag-tagged DDX39B proteins were isolated from the reaction mixtures of in vitro MAP4K2 kinase assays, followed by SDS-PAGE fractionation and trypsin digestion. The trypsin-digested DDX39B peptides were subjected to LC-MS/MS analyses by LTQ-Orbitrap Elite hybrid mass spectrometer. The MASCOT MS/MS Ions Search (Matrix Science) analysis condition: peptide mass tolerance, 20 ppm; fragment MS/MS tolerance, 0.6 Da; allow up to 1 missed cleavage; peptide charge, 2^+^, 3^+^, and 4^+^. Mass spectrometry data are available in a public, open access repository (ProteomeXchange #PXD070831 and #PXD070940).

### Statistics.

Statistical analyses were performed by using Excel 2016, SPSS 25, Partek Flow, or BD SEQGEQ. Two groups were compared by 2-tailed or 1-tailed unpaired Student’s *t* test, as well as Wilcoxon’s rank-sum test. Three or 4 groups were compared by 1-way ANOVA test, followed by Tukey’s post hoc test. The Kruskal-Wallis test was used to analyze violin plot. *P* values less than 0.05 were considered to be significant.

### Study approval.

All animal experiments were approved by the IACUC of National Health Research Institutes (NHRI). All experiments using human clinical samples were approved by the Institutional Review Boards.

### Data availability.

Our scRNA-seq data and mass spectrometry data have been uploaded to a public, open access repository (NCBI SRA #PRJNA990957, ProteomeXchange #PXD070831, and ProteomeXchange #PXD070940). Other data are available upon reasonable request. Values for individual data points in graphs are reported in the Supporting Data Values file.

## Author contributions

HCC performed and supervised experiments, data analysis, data interpretation, study design, and manuscript writing. CWW, CHH, YZX, PMH, CYT, ELT, and HYC performed experiments and analyzed data. THT conceived the study, supervised experiments, interpreted data, and wrote the manuscript.

## Funding support

- National Health Research Institutes (NHRI), Taiwan (IM-113-SP-02 and IM-113-GP01 to THT).
- National Science and Technology Council, Taiwan (NSTC-110-2320-B-400-018 and 113-2320-B-400-017 to THT; NSTC-113-2628-B-400-001-MY3 to HCC).
- National Taiwan University Hospital Hsin-Chu Branch, Taiwan (114-HCH001 and HR-114-H01 to HYC).

## Supplementary Material

Supplemental data

Unedited blot and gel images

Supporting data values

## Version 1 01/30/2026

In-Press Preview

## Version 2 03/16/2026

Electronic publication
