# Supplementary material for: MAP4K2 suppresses antitumor immunity in a pancreatic cancer model by promoting Treg differentiation
Source: J Clin Invest. 2026 Jan 29;136(6):e196379. doi: 10.1172/JCI196379 (PMC12987614; doi:10.1172/JCI196379)
Supplement: Supplemental data [file jci-136-196379-s227.pdf]

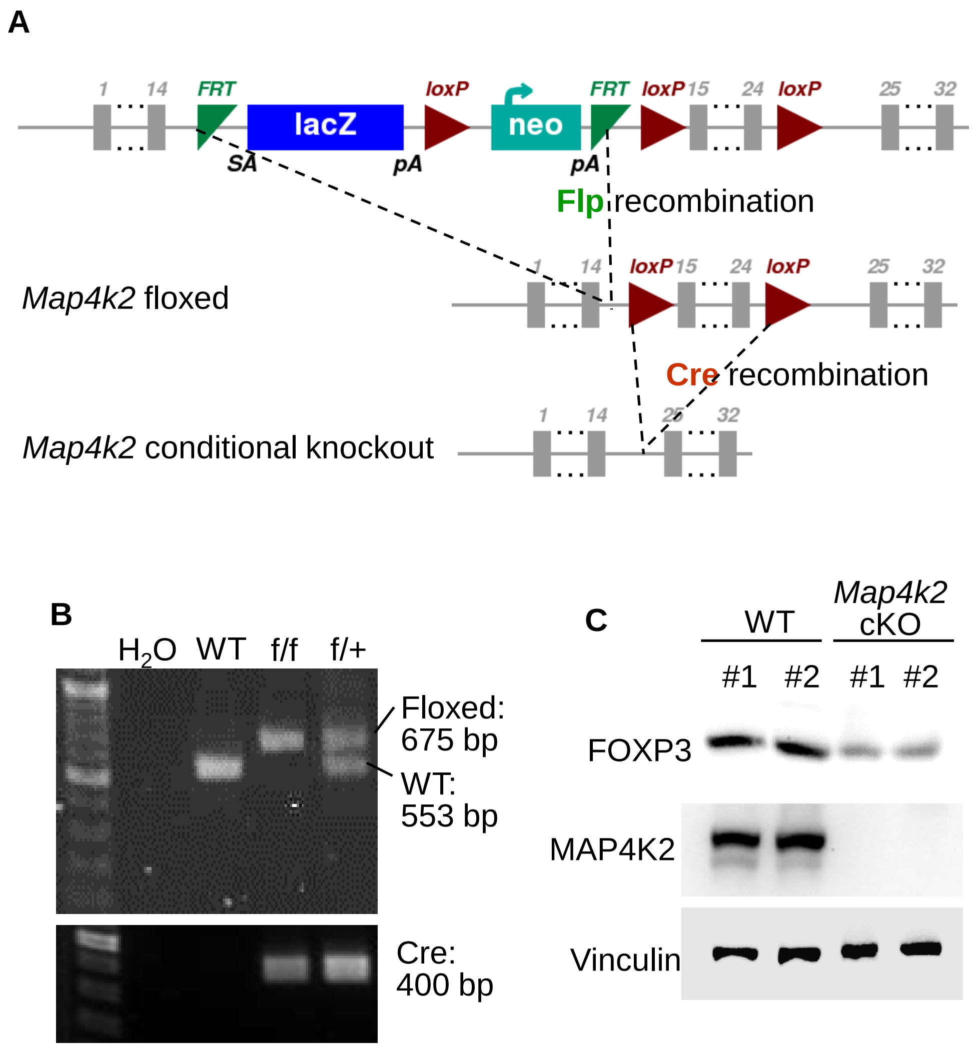

Supplemental Figure 1. Generation of T-cell-specific *Map4k2* conditional knockout mice. (**A**) The schematic diagram of the *Map4k2* knockout (KO) first allele (reporter-tagged insertion with conditional potential) and the resulting floxed *Map4k2* allele. The KO-first allele is initially a non-expressive form, but can be converted to a conditional floxed allele via Flp recombination. (**B**) The PCR product of the 675-bp band indicates the *Map4k2* floxed allele; the 553-bp band indicates the wild-type (WT) allele. (**C**) Immunoblotting analysis of Foxp3 and MAP4K2 protein levels in purified T cells from T-cell-specific *Map4k2* cKO (*Map4k2*$^{f/f}$;*Cd4*-Cre) and wild-type (*Map4k2*$^{f/f}$) mice. Data shown are representative of three independent experiments.

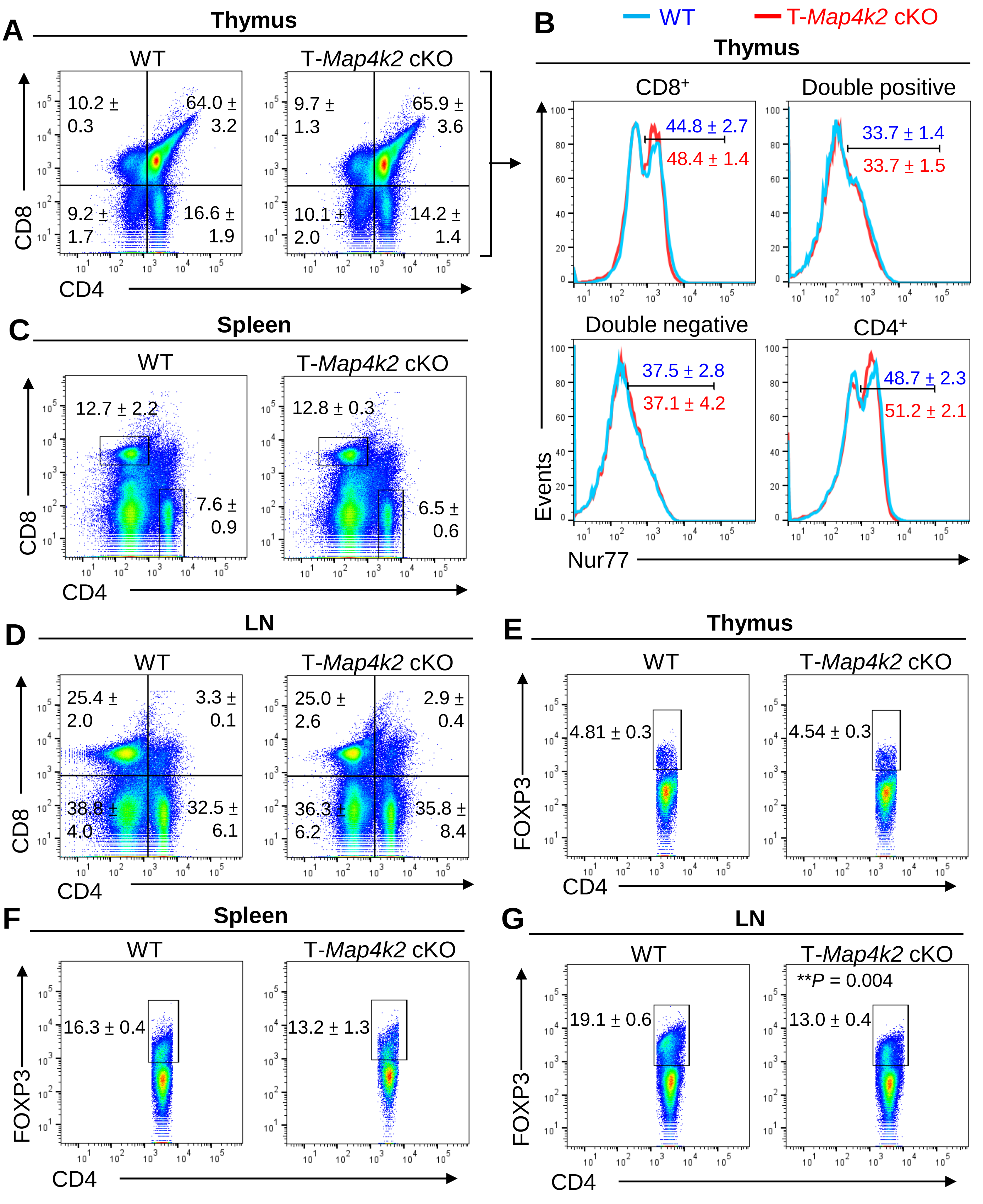

Supplemental Figure 2. Four-week-old T-*Map4k2* cKO mice display reduced Treg population in the spleen and lymph nodes but not the thymus. (**A-G**) Flow cytometry analyses of T cells (A-D) and Treg cells (E-G) from the thymus, spleen, or lymph nodes of 4-week-old T-*Map4k2* cKO or wild-type (WT) mice. T-*Map4k2* cKO, T-cell-specific *Map4k2* conditional knockout (*Map4k2*$^{f/f}$;*Cd4*-Cre); WT, wild-type (*Map4k2*$^{f/f}$). Data shown (mean ± SEM) are representative of three independent experiments.

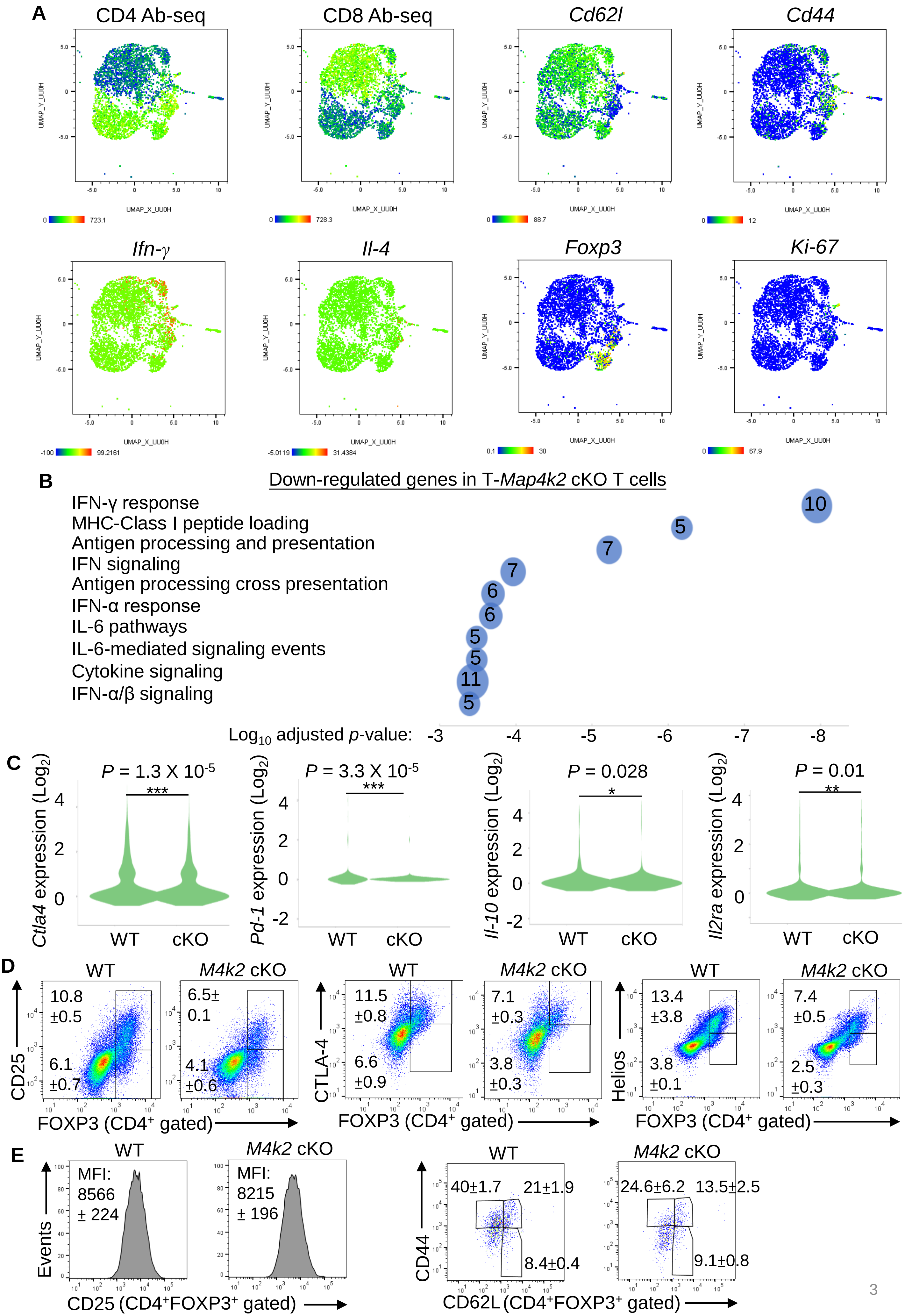
A
CD4 Ab-seq
CD8 Ab-seq
Cd62l
Cd44
Ifn-γ
Il-4
Foxp3
Ki-67
UMAP_X_UU0H
UMAP_Y_UU0H
B
Down-regulated genes in T-Map4k2 cKO T cells
IFN-γ response
MHC-Class I peptide loading
Antigen processing and presentation
IFN signaling
Antigen processing cross presentation
IFN-α response
IL-6 pathways
IL-6-mediated signaling events
Cytokine signaling
IFN-α/β signaling
Log10 adjusted p-value:
C
P = 1.3 X 10-5
P = 3.3 X 10-5
P = 0.028
P = 0.01
Ctla4 expression (Log2)
Pd-1 expression (Log2)
Il-10 expression (Log2)
Il2ra expression (Log2)
WT
cKO
D
WT
M4k2 cKO
CD25
CTLA-4
Helios
FOXP3 (CD4+ gated)
E
MFI: 8566 ± 224
MFI: 8215 ± 196
Events
CD25 (CD4+FOXP3+ gated)
CD44
CD62L (CD4+FOXP3+ gated)

Supplemental Figure 3. Cytokine signaling pathways and Treg markers are reduced in T cells of T-*Map4k2* cKO mice. (**A**) UMAP plots display the expression levels of individual cell makers in splenic and lymph node T cells of wild-type (WT) and T-*Map4k2* cKO mice. Transcript levels are shown in the indicated color scheme. Ab-seq denotes Ab-Oligos (antibody-oligonucleotides). (**B**) Kyoto Encyclopedia of Genes and Genomes (KEGG) pathway enrichment analysis of 34 downregulated genes in unstimulated T cells of T-*Map4k2* cKO mice compared to wild-type (WT) mice. Varied numbers of genes enriched in individual pathways are presented by different diameter sizes and numbers for individual dots. (**C**) mRNA levels of *Ctla4*, *Pd-1*, *Il-10*, and *Il2ra* (*Cd25*) were decreased in T cells of T-*Map4k2* cKO mice. (**D**) Flow cytometry analyses of CD25, CTLA-4, Helios, and FOXP3-expressing $CD4^+$ T cells from the lymph nodes of T-*Map4k2* (*M4k2*) cKO or WT mice. (**E**) Flow cytometry analyses of CD25, CD44, and CD62L-expressing Treg ($CD4^+FOXP3^+$) cells from the lymph nodes of T-*Map4k2* (*M4k2*) cKO or WT mice. T-*Map4k2* cKO, T-cell-specific *Map4k2* conditional knockout (*Map4k2*$^{f/f}$;*Cd4*-Cre); WT, wild-type (*Map4k2*$^{f/f}$); MFI, mean fluorescence intensity. *, P value < 0.05; ***, P value < 0.001 (Kruskal-Wallis test).

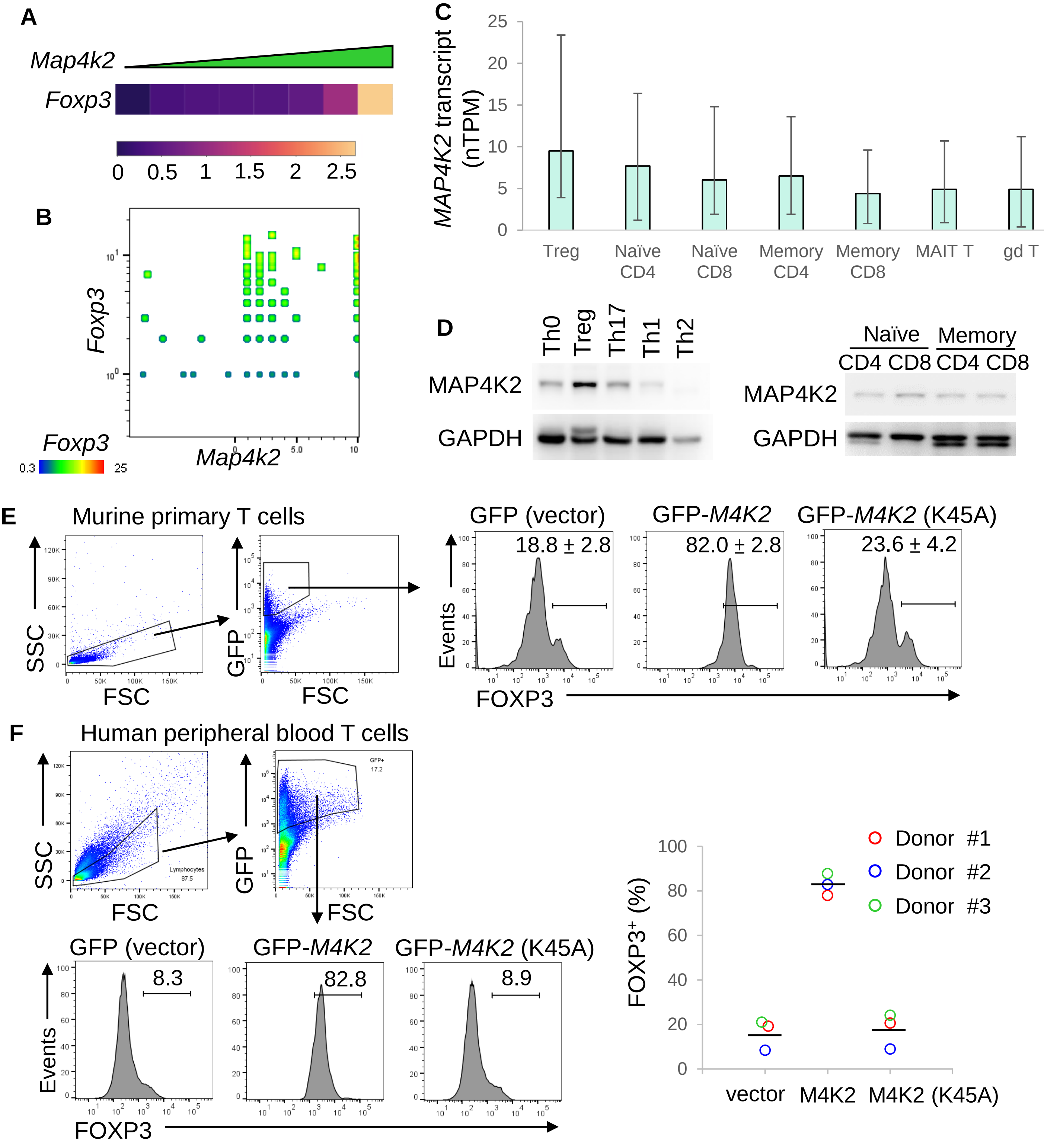

Supplemental Figure 4. MAP4K2 levels are increased in Treg cells. (**A** and **B**) Heatmap (A) and single-cell-expressing plot (B) showing *Foxp3* upregulation in wild-type T cells with concomitantly elevated *Map4k2* expression. (**C**) *MAP4K2* transcript levels in human Treg cells were higher than those of other human T-cell subsets. Data were derived from Human Protein Atlas (HPA) database (https://www.proteinatlas.org/ENSG00000168067-*MAP4K2*/single+cell). N = 109. Data shown are nTPM ± 25th and 75th percentiles. nTPM, normalized transcripts per million. (**D**) Immunoblotting analyses of MAP4K2 and GAPDH proteins in the undifferentiated T cells (Th0) and *in vitro* differentiated Treg/Th17/Th1/Th2 cells derived from splenic T cells of wild-type mice. (**E** and **F**) Flow cytometry analyses of Foxp3 levels in MAP4K2-overexpressing murine primary T cells (E) and human peripheral blood T cells (F). n = 3. T-*Map4k2* cKO, T-cell-specific *Map4k2* conditional knockout (*Map4k2*$^{f/f}$;*Cd4*-Cre); WT, wild-type (*Map4k2*$^{f/f}$).

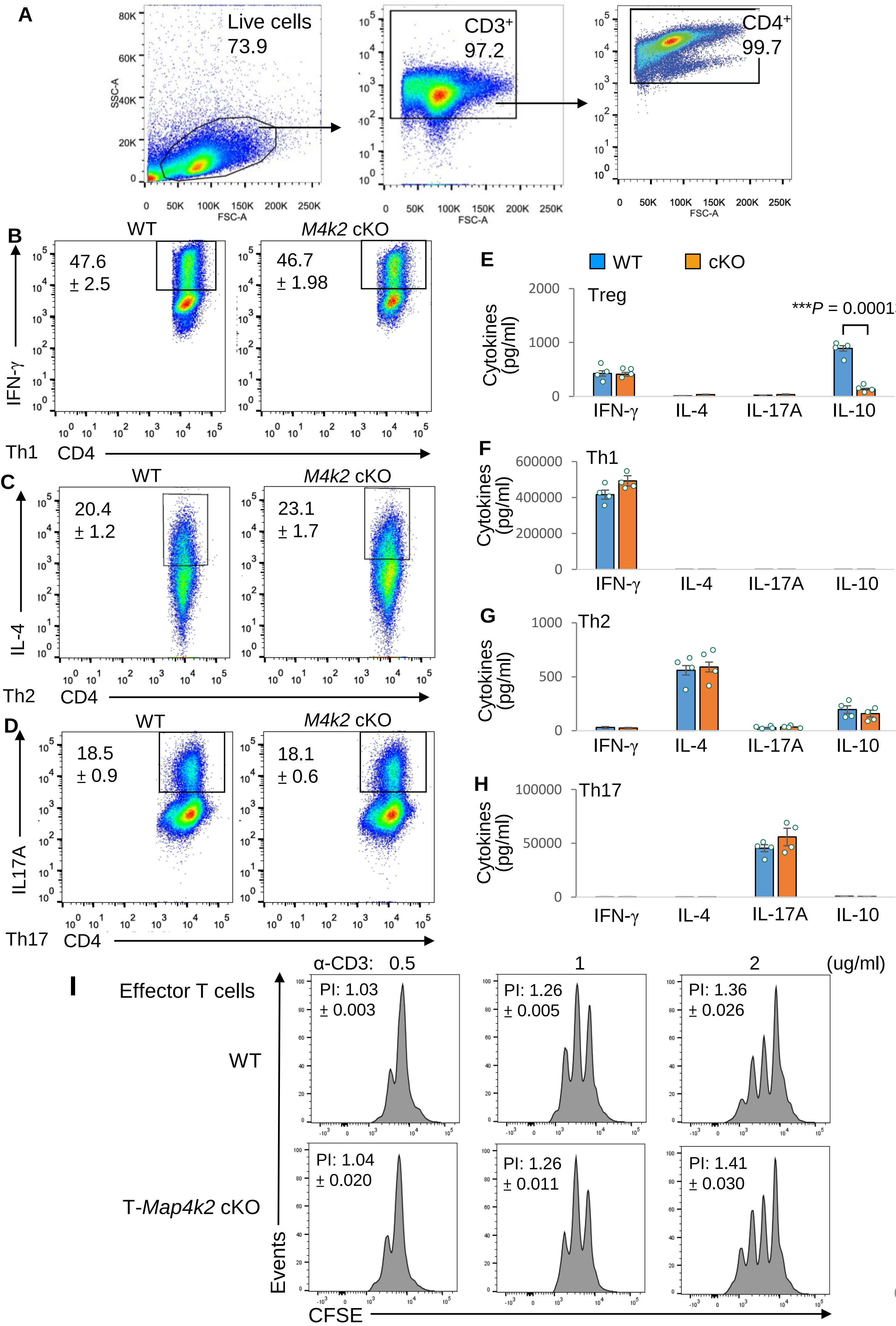
A
Live cells
73.9
SSC-A
FSC-A
CD3+
97.2
CD4+
99.7
B
WT
M4k2 cKO
47.6
± 2.5
46.7
± 1.98
IFN-γ
Th1
CD4
C
WT
M4k2 cKO
20.4
± 1.2
23.1
± 1.7
IL-4
Th2
CD4
D
WT
M4k2 cKO
18.5
± 0.9
18.1
± 0.6
IL17A
Th17
CD4
E
WT
cKO
Treg
***P = 0.00013
Cytokines (pg/ml)
IFN-γ
IL-4
IL-17A
IL-10
F
Th1
Cytokines (pg/ml)
IFN-γ
IL-4
IL-17A
IL-10
G
Th2
Cytokines (pg/ml)
IFN-γ
IL-4
IL-17A
IL-10
H
Th17
Cytokines (pg/ml)
IFN-γ
IL-4
IL-17A
IL-10
I
Effector T cells
α-CD3: 0.5
1
2
(ug/ml)
WT
PI: 1.03
± 0.003
PI: 1.26
± 0.005
PI: 1.36
± 0.026
T-Map4k2 cKO
PI: 1.04
± 0.020
PI: 1.26
± 0.011
PI: 1.41
± 0.030
Events
CFSE

Supplemental Figure 5. Th1, Th2, or Th17 *in vitro* differentiation and T-cell proliferation are unaffected by *Map4k2* conditional knockout. (**A-D**) Flow cytometry analyses of Th1 ($CD4^{+}IFN\text{-}\gamma^{+}$) (B), Th2 ($CD4^{+}IL\text{-}4^{+}$) (C), Th17 ($CD4^{+}IL\text{-}17A^{+}$) cells of in vitro differentiated T cells using splenic T cells from wild-type (WT) mice or T-*Map4k2* (*M4k2*) cKO mice. (**E-H**) ELISAs of individual cytokines in the supernatants of in vitro differentiated T cells using splenic T cells from WT mice or T-*Map4k2* cKO mice. (**I**) CFSE dilution assays of TCR-induced T cell proliferation using effector T cells of WT or T-*Map4k2* mice. Means ± SEM are shown. T-*Map4k2* cKO, T-cell-specific *Map4k2* conditional knockout (*Map4k2*$^{f/f}$;*Cd4*-Cre); WT, wild-type (*Map4k2*$^{f/f}$); PI, proliferation index. Data shown are representative of three independent experiments.

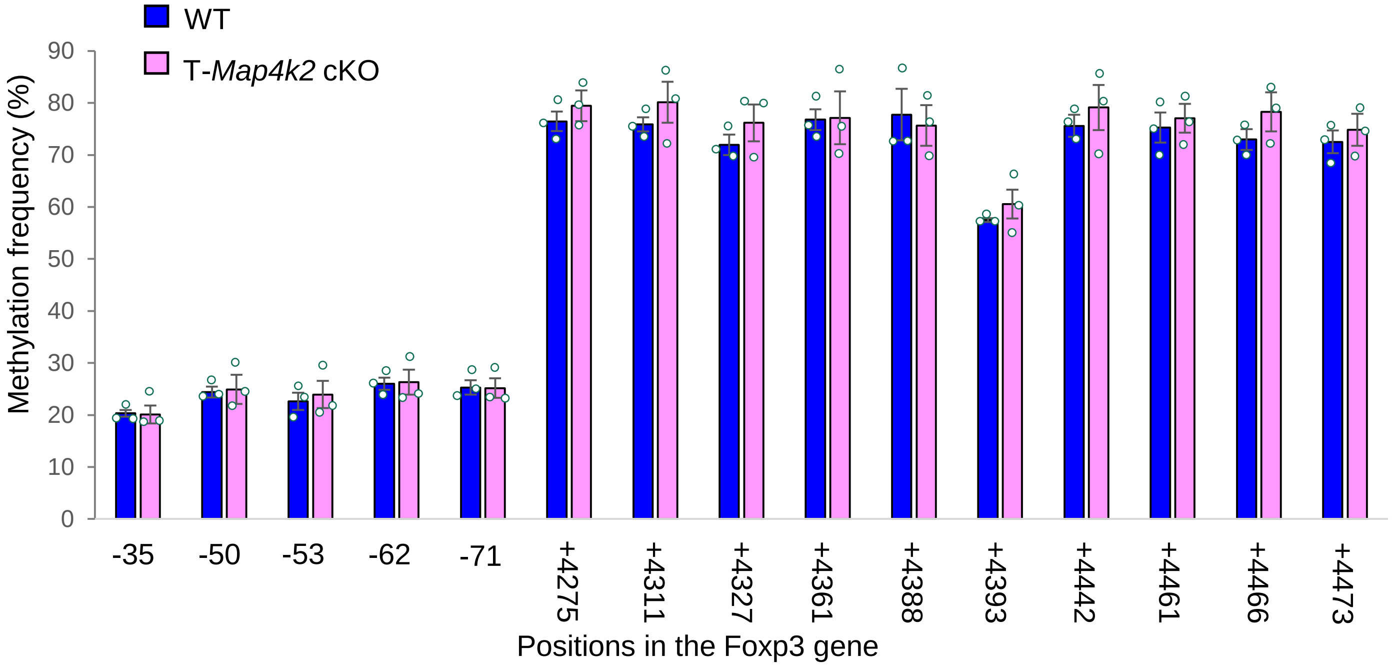

Supplemental Figure 6. The methylation of Treg-specific demethylated region is normal in Treg cells of T-*Map4k2* cKO mice. The methylation frequencies of Treg-specific demethylated region (TSDR) were determined by pyrosequencing using Treg cells isolated from the spleen and lymph nodes of wild-type (WT) mice or T-*Map4k2* cKO mice. n = 3. Means ± SEM are shown. T-*Map4k2* cKO, T-cell-specific *Map4k2* conditional knockout (*Map4k2*$^{f/f}$;*Cd4*-Cre); WT, wild-type (*Map4k2*$^{f/f}$).

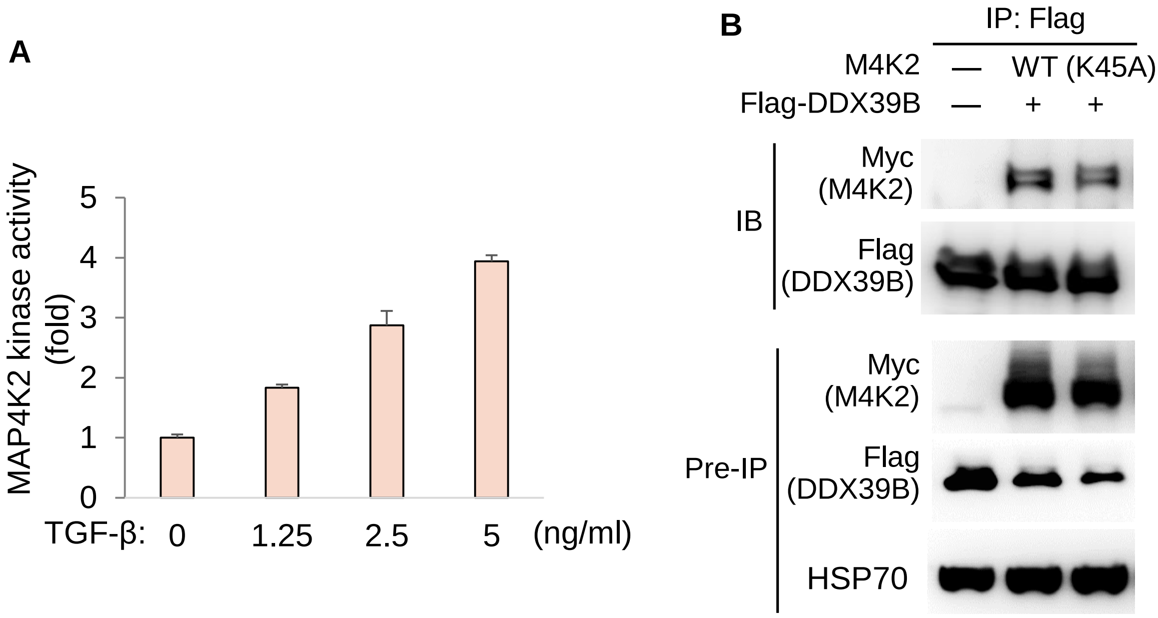

Supplemental Figure 7. MAP4K2 kinase activity is induced by TGF-β stimulation. (**A**) In vitro kinase assays of purified MAP4K2 proteins isolated from the lysates of Jurkat T cells transfected with Flag-*MAP4K2* plasmid. The transfected Jurkat T cells were stimulated with TGF-β for 30 min. n = 2. Results (mean ± SD) are presented relative to the unstimulated control. (**B**) Co-immunoprecipitation of Flag-tagged DDX39B with either Myc-tagged MAP4K2 or MAP4K2 (K45A) mutant proteins from the lysates of HEK293T cells co-transfected with Flag-*DDX39B* plasmid plus either Myc-*MAP4K2* or Myc-*MAP4K2* (K45A) plasmid. WT, wild-type MAP4K2 (M4K2); K45A, MAP4K2 kinase-dead mutant.

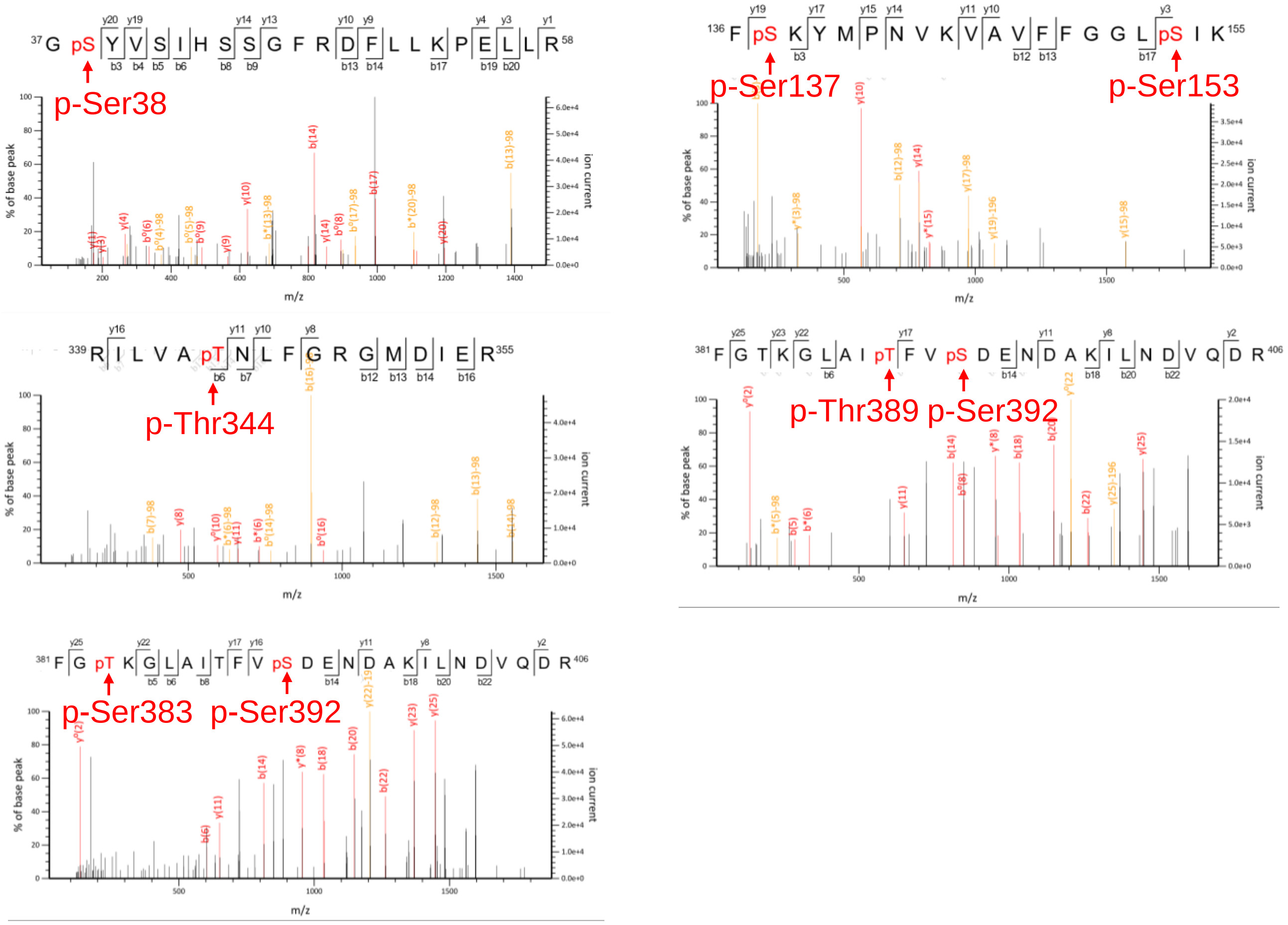

Supplemental Figure 8. Phosphorylated peptides of DDX39B protein in the MAP4K2 immunocomplexes are detected by mass spectrometry analysis. Mass spectrometry analysis of the DDX39B peptides containing phosphorylation residues. Data show phosphorylated Ser38, Ser137, Ser153, Thr344, Thr383, Thr389, and Ser392 residues of DDX39B proteins in the MAP4K2 immunocomplexes isolated from Jurkat T cells stimulated with anti-CD3 antibody (5 µg/ml for 10 min).

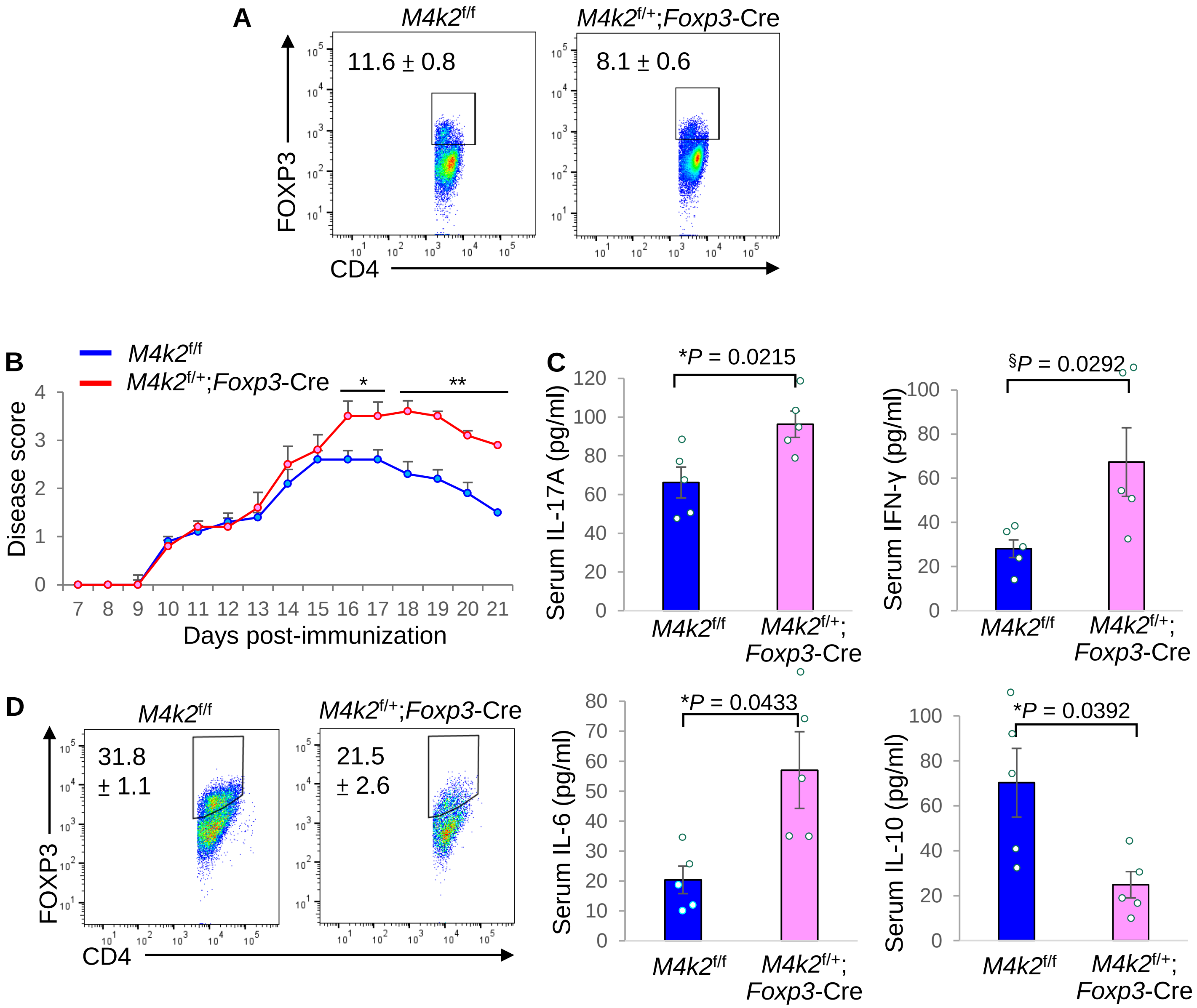

Supplemental Figure 9. Treg-specific *Map4k2*-deficient mice display sustained induction of EAE autoimmune inflammation. (**A**) Flow cytometry analysis of Treg cells in the spleen of mice without immunization. (**B-D**) Induction of MOG-induced experimental autoimmune encephalomyelitis (EAE) using Treg-specific *Map4k2*-deficient mice or WT mice. Clinical scores of diseased mice are shown in mean ± SEM (A, n = 5). The cytokine levels in the sera of diseased mice on day 21 of the MOG-induced EAE model were determined using ELISA assays (B, mean ± SEM, n = 5). Infiltrating Treg cells in the brain of diseased mice on day 21 were determined by flow cytometry (C, n = 5). WT, wild-type (*Map4k2*$^{f/f}$). *M4k2*$^{f/+}$;*Foxp3*-Cre, Treg-specific *Map4k2*-deficient mice. §, *P* value < 0.05; (one-tailed Student's t-test); *, *P* value < 0.05; **, *P* value < 0.01 (two-tailed Student's t-test).

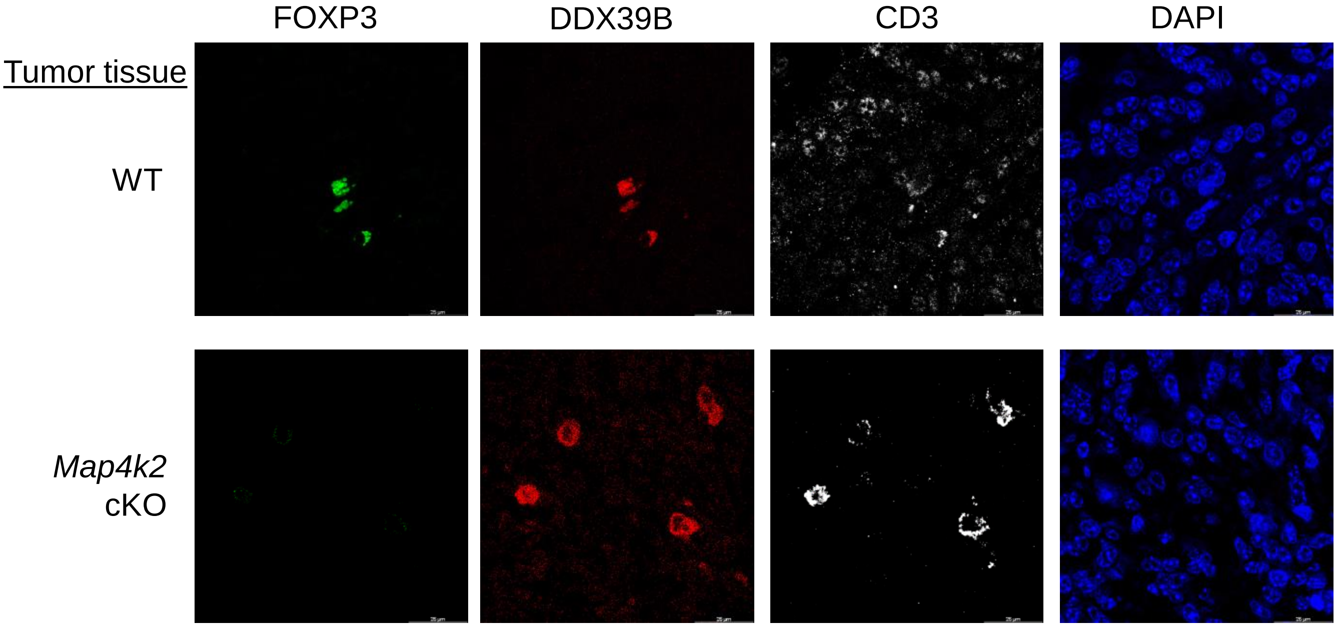

Supplemental Figure 10. DDX39B proteins are mainly localized in the cytoplasm of *Map4k2* cKO T cells. Confocal microscopy analyses of FOXP3 (green), DDX39B (red), CD3 (gray), and DAPI (blue) in the tumor tissues from tumor-bearing WT and T-*Map4k2* cKO mice on day 16 of the syngeneic KPC pancreatic cancer model.

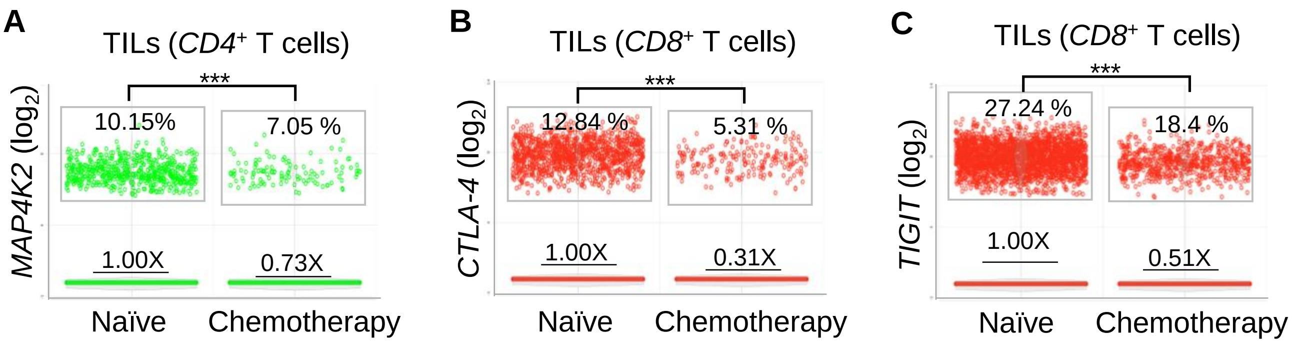

Supplemental Figure 11. *MAP4K2* levels in tumor-infiltrating *CD4*$^{+}$ T cells are decreased after chemotherapy. (A-C) Analysis of single-cell RNA sequencing dataset derived from 20 pancreatic cancer untreated patients or 7 patients with a chemotherapy treatment. Plots show *MAP4K2* levels in the tumor-infiltrating *CD4*$^{+}$ T cells (A), *CTLA-4* levels in *CD8*$^{+}$ T cells (B), and *TIGT* levels in *CD8*$^{+}$ T cells (C) from untreated patients or patients treated with a chemotherapy. TILs, tumor-infiltrating lymphocytes.
