# Supplementary material for: MAP4K2 suppresses antitumor immunity in a pancreatic cancer model by promoting Treg differentiation
Source: J Clin Invest. 2026 Jan 29;136(6):e196379. doi: 10.1172/JCI196379 (PMC12987614; doi:10.1172/JCI196379)

# Figure 3B

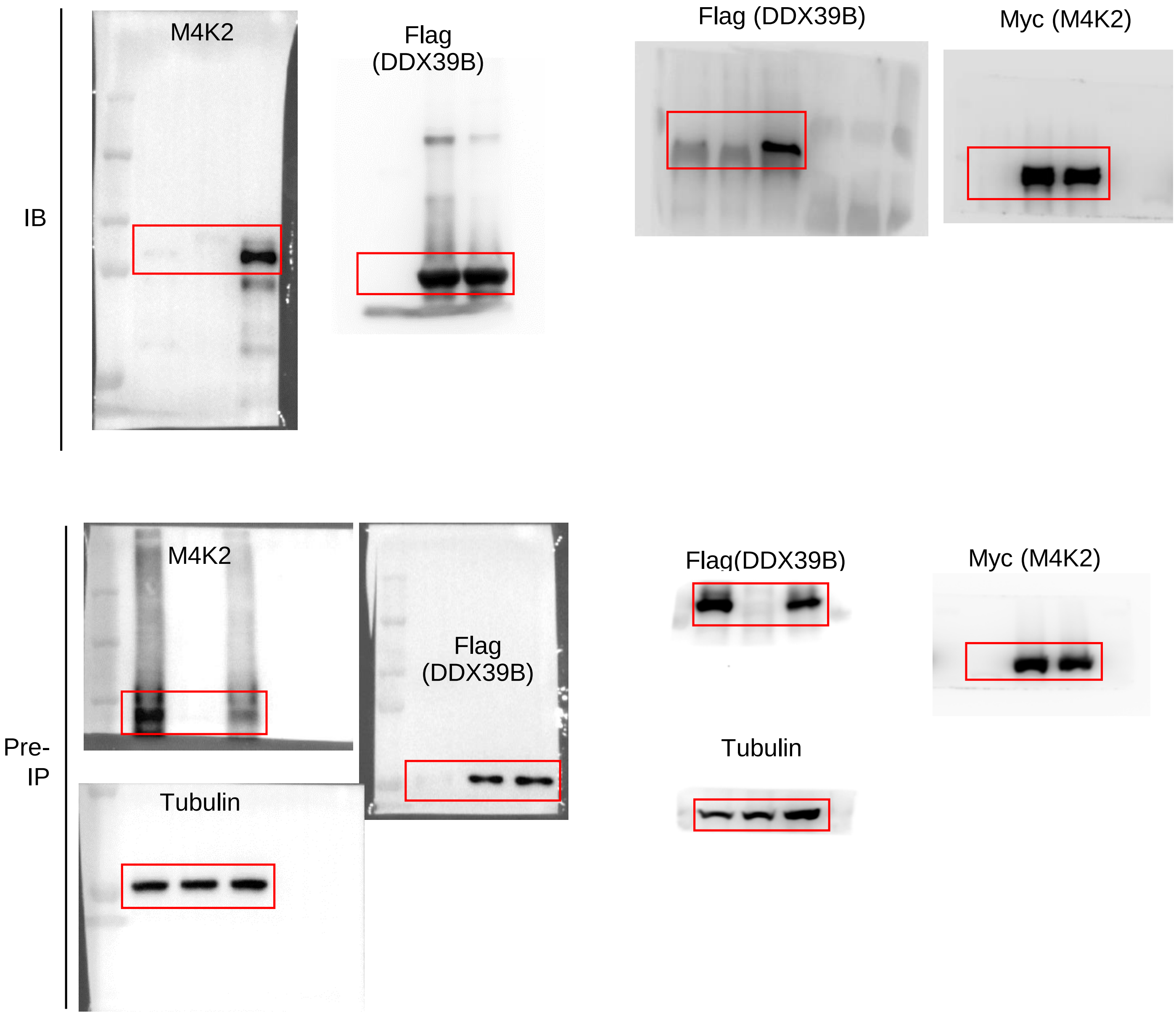


# Figure 3E

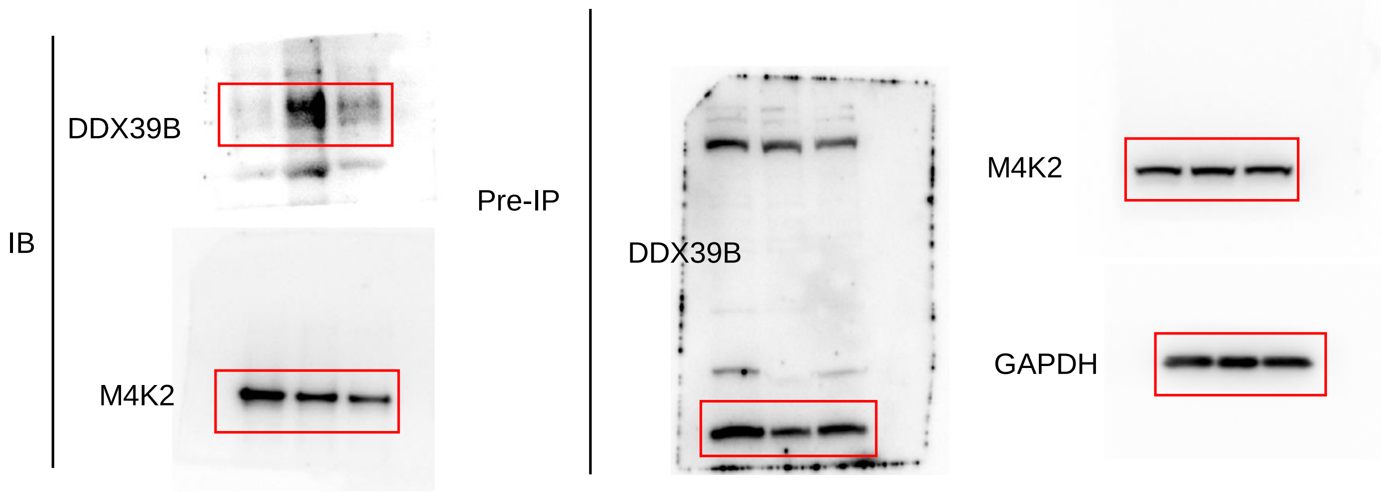

**Figure 5B**

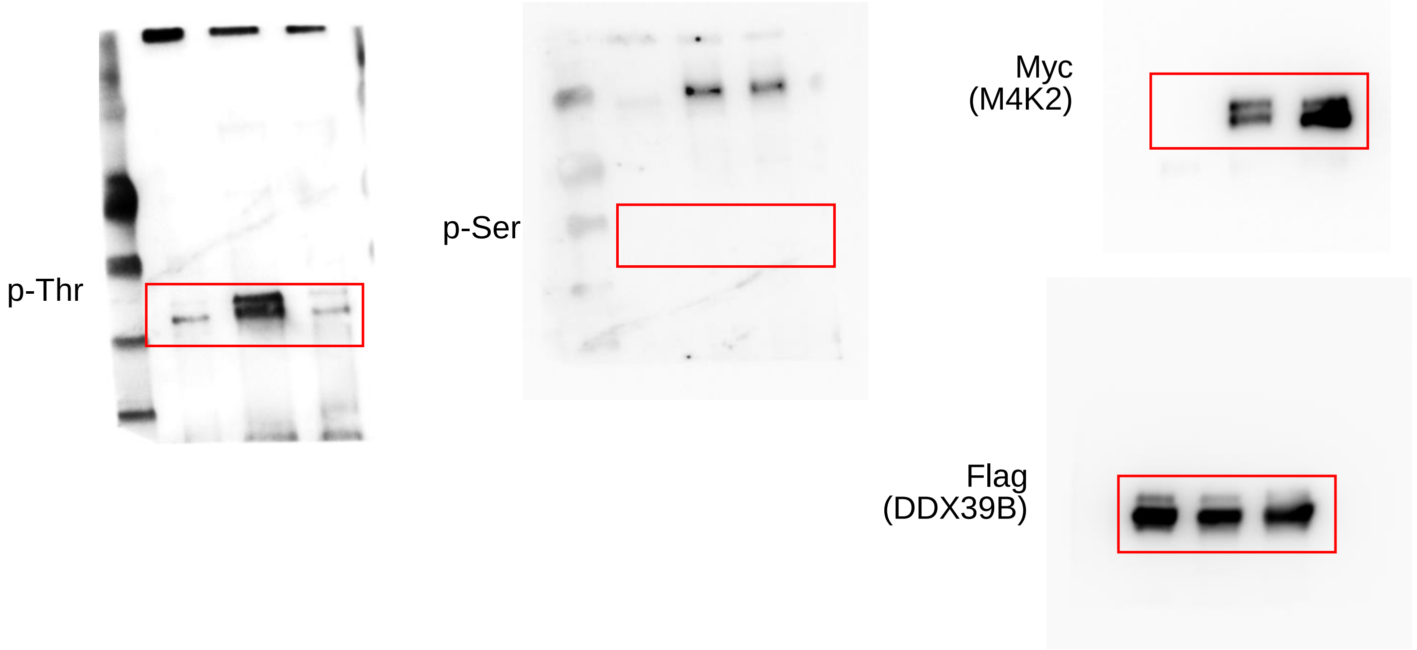


**Figure 6B**

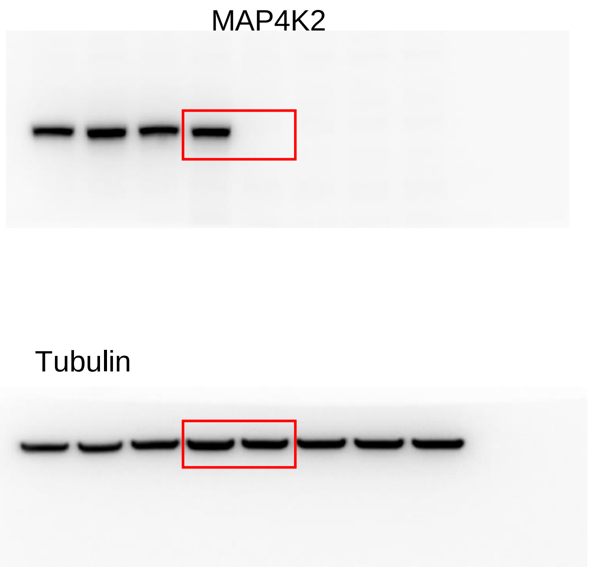


**Figure 9D**

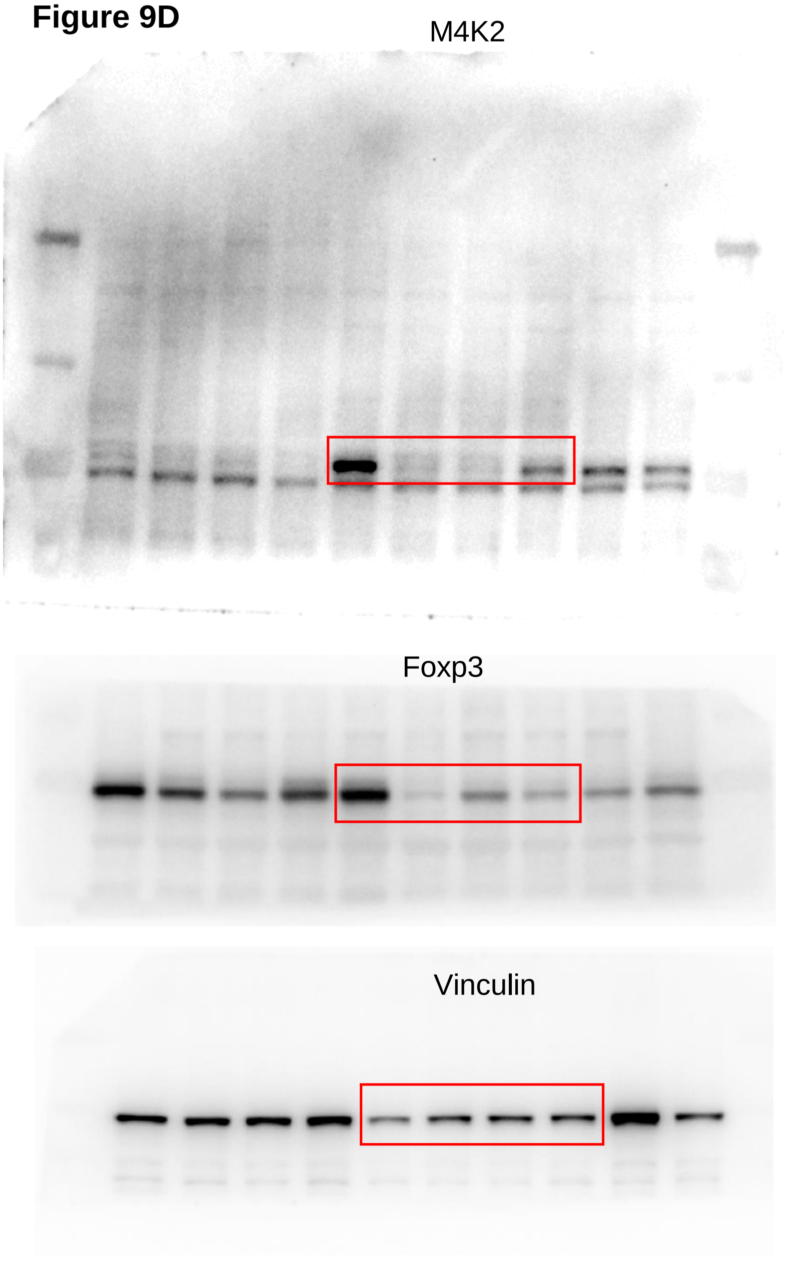

**Figure 10C**

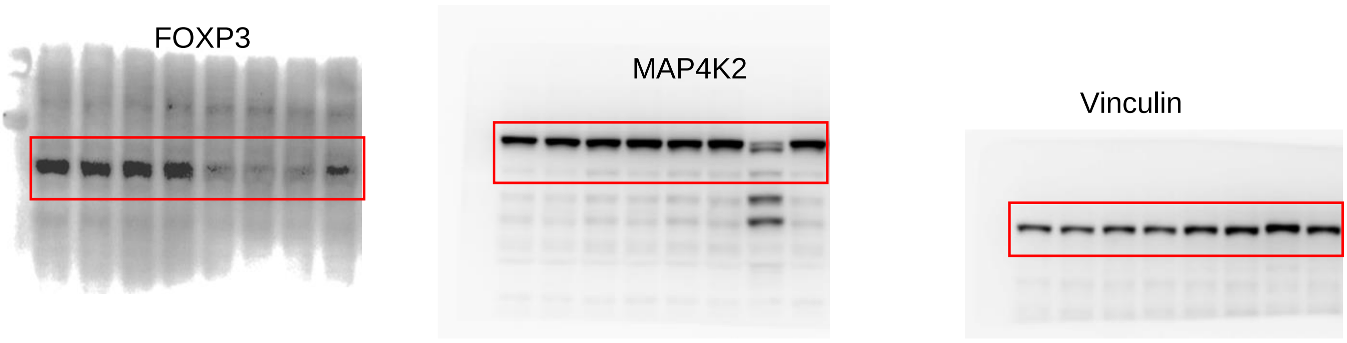


**Supplemental Figure 1C**

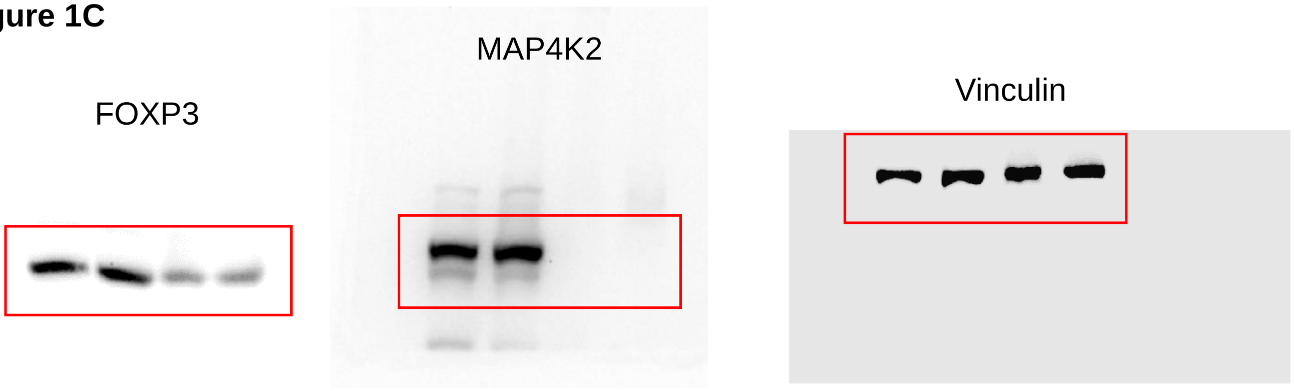


**Supplemental Figure 4D**

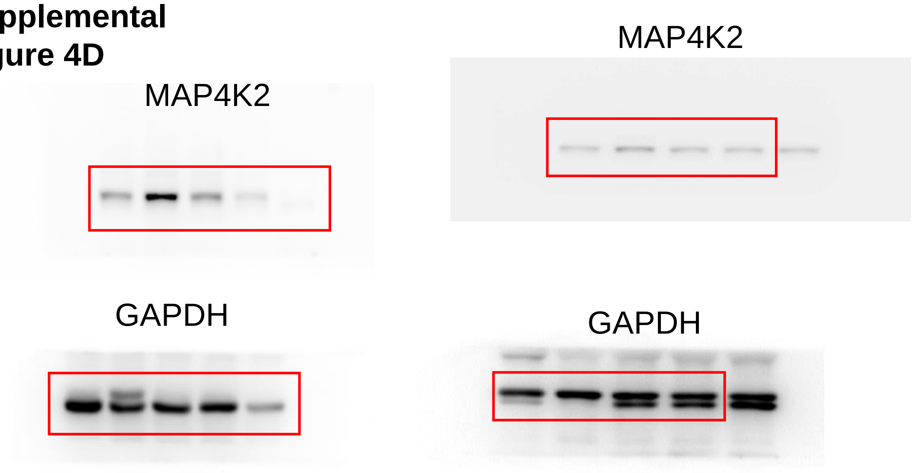

**Supplemental Figure 7B**

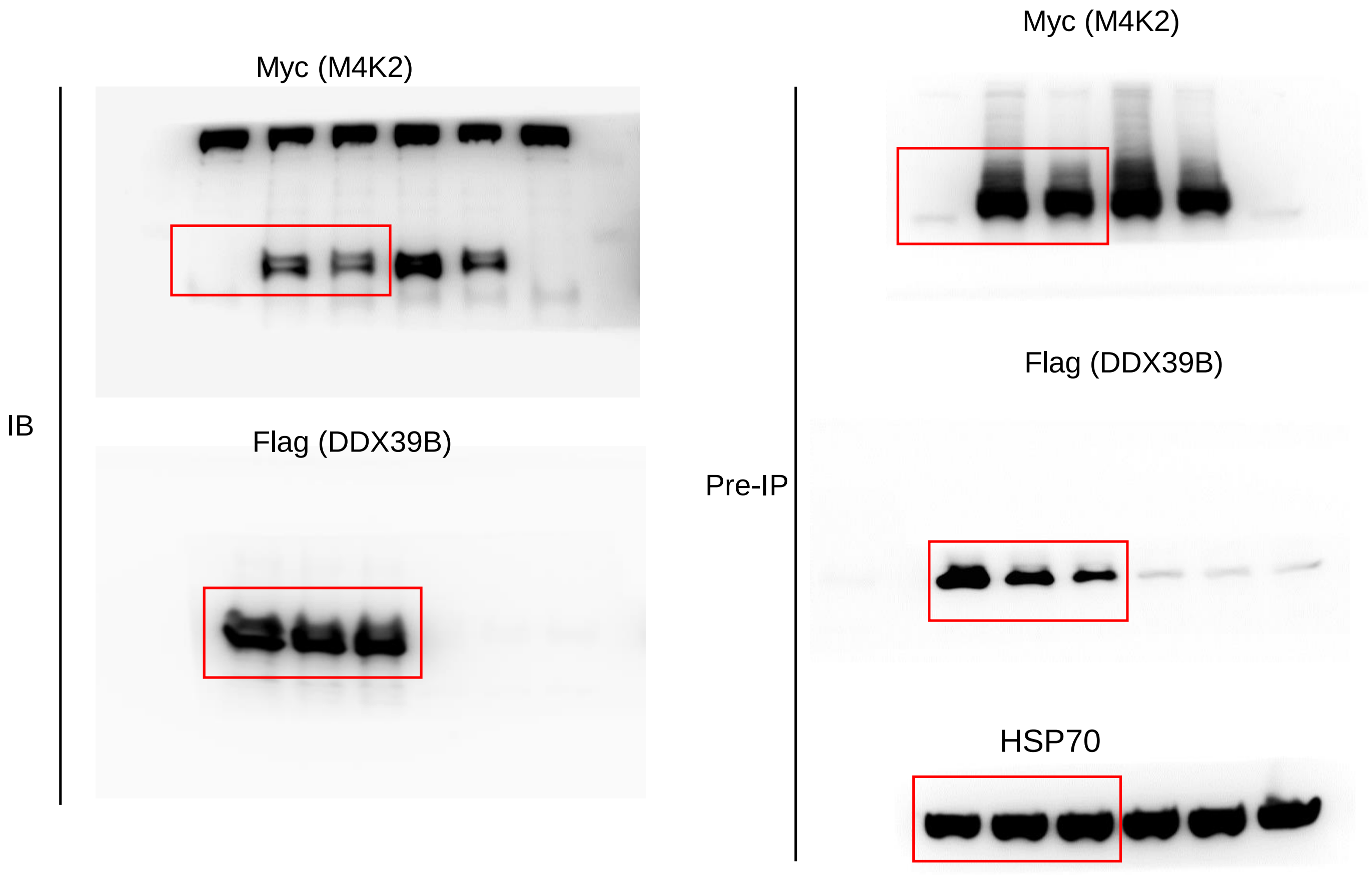

Supplement: Unedited blot and gel images [file jci-136-196379-s228.pdf]
